# Organic substrate oxidation-driven water electrolysis enabled by Ru-loaded Mn_2_O_3_ nanoparticles for energy-efficient hydrogen generation

**DOI:** 10.1039/d6na00300a

**Published:** 2026-09-14

**Authors:** Sachin P. D., Ashoka S, Madesh Kumar M, Yogesh K.

**Affiliations:** a Department of Chemistry, School of Applied Sciences, REVA University Bengaluru-560064 India ashok022@gmail.com; b Center for Nano & Functional Materials, REVA Research Center, REVA University Bengaluru-560064 India; c Department of Physics, School of Applied Sciences, REVA University Bengaluru-560064 India; d Department of Physics, School of Engineering, Dayananda Sagar University Bengaluru South-562112 India

## Abstract

Transforming the inefficient oxygen evolution reaction (OER) by integrating beneficial organic substrate oxidation reactions offers an innovative way to significantly lower the energy requirements of water electrolysis while simultaneously producing valuable chemicals. This study introduces a ruthenium-loaded manganese oxide electrocatalyst (Ru-Mn_2_O_3_-U), synthesized through a rapid urea-assisted redox method. This approach not only ensures an even distribution of ruthenium, preventing harmful particle densification, but also creates easily accessible active centers on the surface, thereby enhancing both mass and charge transfer kinetics. The Ru-Mn_2_O_3_-U catalyst demonstrates exceptional performance, achieving current densities of 100 mA cm^−2^ and 400 mA cm^−2^ at potentials of 1.32 V and 1.45 V *versus* the reversible hydrogen electrode (RHE) during the ethylene glycol electrooxidation reaction (EGEOR). With a specific mass activity of 108.4 A g^−1^ and a surface-specific activity of 0.325 mA cm^−2^, it demonstrates an impressive faradaic efficiency of 91.8% by primarily converting ethylene glycol (EG) into glycolate. In practical applications, the EG-assisted electrolyzer, comprising a Ru-Mn_2_O_3_-U‖Ru-Mn_2_O_3_-U cell, reaches a current density of 100 mA cm^−2^ at just 1.54 V, showcasing a hydrogen faradaic efficiency of around 93% along with good durability. By effectively combining hydrogen production with the oxidation of biomass-derived alcohols, this approach enhances the energy efficiency of electrochemical hydrogen generation, paving the way for a sustainable energy future.

## Abstract 2

Integrating the ethylene glycol oxidation reaction catalyzed by Ru-Mn_2_O_3_ to replace the oxygen evolution reaction reduces the energy requirements for hydrogen production while simultaneously producing valuable formate.

## Graphical abstract

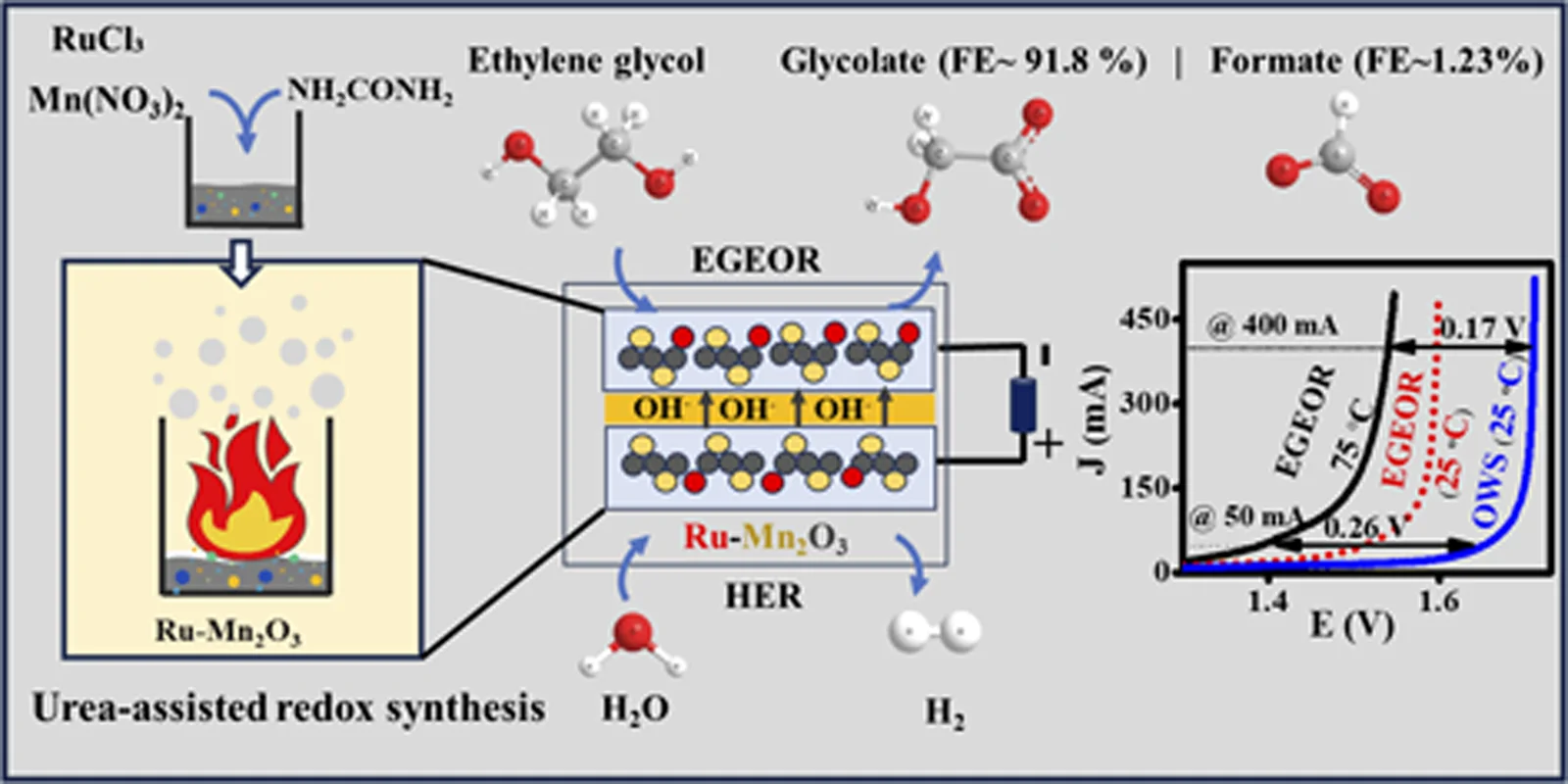

## 1 Introduction

The urgent need for sustainable electrochemical energy conversion and storage devices is driving a surge in research interest. As global energy demand continues to rise and the detrimental effects of carbon-based fossil fuels become increasingly evident, innovative solutions are essential for a cleaner, more sustainable future.^1^ This focus has spurred research into efficient hydrogen production, which offers high energy density and a carbon-free combustion product, making it a promising alternative to fossil fuels for power generation, transportation, and industrial processes.^2^ Electrochemical water splitting is particularly noteworthy as a sustainable method of producing ultra-pure hydrogen.^3^ However, conventional water splitting approaches face challenges, including high energy consumption and limited economic value from the oxygen produced during the anodic OER.^4^ These issues have driven researchers to seek alternative anodic reactions to reduce energy demand and generate higher-value chemicals.

Electrooxidation reactions (EOR) of organic substrates are promising alternatives to the OER in water splitting, as they operate at lower potentials, significantly reducing the energy needed for hydrogen generation.^5,6^ The EOR reduces energy demands while producing high-value chemicals, making it a promising method for efficient hydrogen production.^7,8^ In particular, the EOR of alcohols and polyols has garnered interest due to their favorable thermodynamics, adjustable reaction pathways, and connection to biomass-derived feedstocks.^9–14^ EG is an attractive anodic substrate for water electrolysis due to its low thermodynamic potential, high availability, low toxicity, low volatility, and ease of storage and transportation.^15^ The electrooxidation of EG takes place at a lower cell potential than water electrolysis, significantly lowering the energy consumption for hydrogen production.^16^ The EGEOR generates crucial chemicals, including formic acid and glycolic acid, along with various other products that depend on the chosen reaction pathway.^17,18^ Glycolate is a valuable compound with diverse applications across industries. In textiles, it functions as a dyeing and tanning agent. In food, it enhances flavor and acts as a preservative. In pharmaceuticals, it serves as an effective skincare agent. Its versatility makes glycolate an essential asset in the market.^19^ Achieving high selectivity towards glycolate is of great interest for effectively coupling hydrogen production with the EGEOR.

The coupling of the EGEOR with water electrolysis faces several challenges. The EGEOR involves complex multielectron transfer processes, often resulting in poor electrocatalytic performance and low selectivity due to various intermediate products.^20^ Additionally, surface poisoning from strong adsorption of intermediates can hinder catalyst performance. Although precious metal based electrocatalysts are often used for the EGEOR in alkaline electrolytes,^21–24^ their high price, low availability, and vulnerability to poisoning hinder their practical application. Therefore, there is a need for earth-abundant alternative electrocatalysts. Recently, our group demonstrated the effectiveness of cobalt vanadate nanosheets and NiS nanoparticles for the EGEOR coupled with water electrolysis.^25,26^ These catalysts showed better performance at low EG concentrations (around 10–50 mmol), although low concentrations can lead to slow reaction rates and decreased energy efficiency.

Manganese oxides have diverse crystal structures and oxidation states, allowing them to effectively interact with oxygenated species, making them suitable electrocatalysts for alcohol oxidation.^27^ Mn_2_O_3_ is particularly used in electrochemical applications due to its favorable electronic structure and alkaline stability.^28^ However, its poor electrical conductivity and limited electrochemical performance require improvements. Incorporating trace amounts of noble metals into Mn_2_O_3_ matrices offers an effective solution to existing limitations. Ruthenium-based electrocatalysts show excellent performance and favorable adsorption for alcohol substrates.^29–31^ Combining ruthenium with metal oxides enhances alcohol electrooxidation kinetics by improving charge transfer and adsorption energies. Low concentrations of ruthenium in the metal oxide matrix maximize efficiency while reducing noble metal usage. These interactions boost activity and selectivity in various alcohol oxidation reactions, highlighting the potential of ruthenium metal oxide hybrid electrocatalysts for the EGEOR.^32–34^ Synthesis methods greatly impact the performance of electrocatalysts. Achieving a uniform dispersion of active sites and optimal contact between components is essential for improving electrocatalytic efficiency. The urea-assisted redox synthesis method offers a quick, one-pot approach to produce mixed metal oxide electrocatalysts with uniform elemental distribution. This process involves an exothermic reaction between metal nitrates and urea, resulting in nanocrystalline materials with enhanced active sites at the surface and improved electrochemical activity.

This study presents a synthesis of ruthenium-loaded Mn_2_O_3_ to replace the sluggish OER with the EGEOR for efficient hydrogen generation. The method ensures a uniform distribution of ruthenium in the Mn_2_O_3_ matrix. The Ru-Mn_2_O_3_-U electrode shows significantly enhanced mass and specific activities, indicating effective use of active ruthenium sites. We also demonstrate the catalyst's practical application with an EG-assisted Ru-Mn_2_O_3_-U‖Ru-Mn_2_O_3_-U electrolyzer, achieving high current densities at low cell potentials and high faradaic efficiency for hydrogen and glycolate production. These findings advance organic substrate oxidation-assisted water electrolysis for sustainable energy conversion.

## 2 Experimental section

### 2.1 Synthesis Mn_2_O_3_ and Ru-loaded Mn_2_O_3_

Manganese nitrate and ruthenium chloride were purchased from Sigma-Aldrich, while ethanol, ethylene glycol, potassium hydroxide pellets, and hydrochloric acid were procured from Sd Fine Chemicals. Nickel foam was procured from Vritra Technologies.

The synthesis of Mn_2_O_3_ is detailed in our recently published research article, and this method has been adapted to prepare Ru-loaded Mn_2_O_3_.^35^ In summary, precise amounts of ruthenium chloride, manganese nitrate tetrahydrate, and urea were combined in 10 ml of distilled water. This solution was then heated at 500 °C for 45 minutes under an air atmosphere in a muffle furnace at a heating rate of 10 °C min^−1^. After the heating process, the beaker was removed and allowed to cool to room temperature before the contents were collected. The Mn_2_O_3_ and Ru-loaded Mn_2_O_3_ synthesized using urea are labeled as Mn_2_O_3_-U and Ru-Mn_2_O_3_-U, respectively. In contrast, Ru-loaded Mn_2_O_3_ synthesized without urea is designated simply as Ru-Mn_2_O_3_. The phase structure, surface chemical composition, and morphology of the prepared samples were analyzed using various techniques, with details provided in the supporting information.

### 2.2 Electrochemical test

The working electrode was created by coating Ru-Mn_2_O_3_ nanoparticles onto nickel foam (1 cm^2^). An electrocatalyst suspension was made by suspending Ru-Mn_2_O_3_ nanoparticles in 200 µL of a 1 : 1 ethanol–water mixture using ultrasonic techniques. This suspension was then coated onto nickel foam, resulting in a mass loading of approximately 3 mg cm^−2^. The electrode made from Mn_2_O_3_ derived from urea is labeled as Mn_2_O_3_-U/NF, while the electrodes fabricated with Ru-Mn_2_O_3_, prepared with and without urea, are respectively labeled as Ru-Mn_2_O_3_-U/NF and Ru-Mn_2_O_3_/NF.

Alkaline electrochemical water-splitting reactions were performed in a solution of 6 M KOH and 6 M KOH containing EG. Ru-Mn_2_O_3_-U/NF, Ag/AgCl, and platinum wire functioned as the working, reference, and counter electrodes, respectively. An electrolyte volume of 15 ml of 6 M KOH was used in a three-electrode configuration. Linear sweep voltammetry (LSV) curves were obtained at a scan rate of 10 mV s^−1^. The electrochemical impedance spectra (EIS) were recorded over a frequency range of 0.01 Hz to 100 kHz. Tafel slopes were determined from the LSV data. Cyclic voltammetry (CV) curves were recorded in a non-faradaic region at scan rates of 70, 90, 110, 130, and 150 mV s^−1^ to calculate the double-layer capacitance (*C*_dl_) and electrochemically active surface area (ECSA). A straight line resulting from the plot of Δ*J vs.* scan rate was used to determine *C*_dl_. The ECSA was calculated using the empirical formula ECSA = *C*_dl_/0.04.^36^ The stability of the Ru-Mn_2_O_3_-U/NF electrode was assessed through chronoamperometry. A membrane-less single-compartment alkaline electrolyzer was assembled with an active electrode area of 1 cm^2^. Furthermore, an alkaline water electrolyzer with a single stack and an active electrode area of 2.2 cm^2^ was constructed. Both electrolyzers are evaluated for their water-splitting performance at temperatures of 298 K and 348 K, both in the presence and in the absence of EG. Additionally, 15 ml and 100 ml of electrolyte were utilized in membrane-less conventional electrolyzers and stacked electrolyzers, respectively.

## 3 Results and discussion

### 3.1 Structure, morphology, and composition analysis

**Fig. 1:**
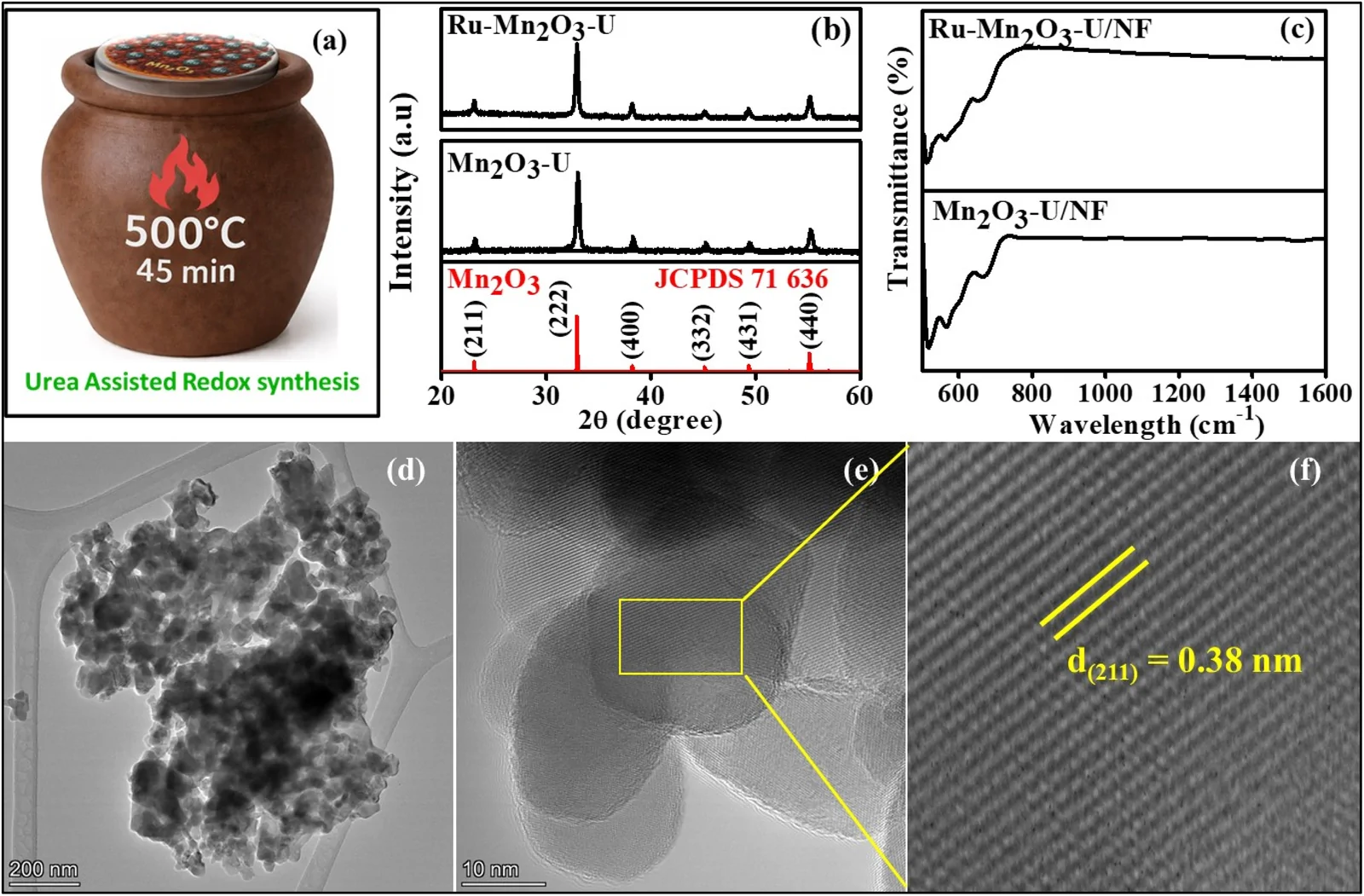
(a) One-pot synthesis; (b) powder XRD patterns; (c) FTIR spectra of Mn_2_O_3_-U and Ru-Mn_2_O_3_-U; (d) TEM image; and (e and f) HRTEM images of Ru-Mn_2_O_3_.

The reaction between an oxidizer and fuel typically generates a very high temperature and significant exothermicity when the oxidizer-to-fuel stoichiometric ratio is 1 in a fuel-assisted redox reaction.^35^ Thus, the fuel-assisted redox process is recognized for its ability to produce highly crystalline and pure oxide nanomaterials in a cost-effective and energy-efficient manner, often within a very short timeframe of just minutes.^37^ Manganese nitrate (oxidizer) and urea (fuel) are reacted in a 1 : 1 ratio, with 0.1% ruthenium chloride, mixed at the molecular level in an aqueous solution. Subsequently, thermal treatment is performed at 500 °C for 45 minutes, leading to the formation of homogeneously distributed ruthenium within Mn_2_O_3_, as illustrated in Fig. 1a. The powder XRD patterns of the product prepared with and without ruthenium (Fig. 1b) feature sharp peaks corresponding to the pure cubic phase of Mn_2_O_3_, which has the *Ia*3̄ space group [JCPDS 71 636].

The presence of cubic Mn_2_O_3_ in both samples is further confirmed by the FTIR spectrum shown in Fig. 1c. The peaks at 517 cm^−1^ and 673 cm^−1^ are ascribed to the Mn–O stretching mode and the Mn–O–Mn asymmetric stretching mode, respectively.^38^ The peak appearing at 566 cm^−1^ indicates the presence of an α-Mn_2_O_3_ structure.^39^

**Fig. 2:**
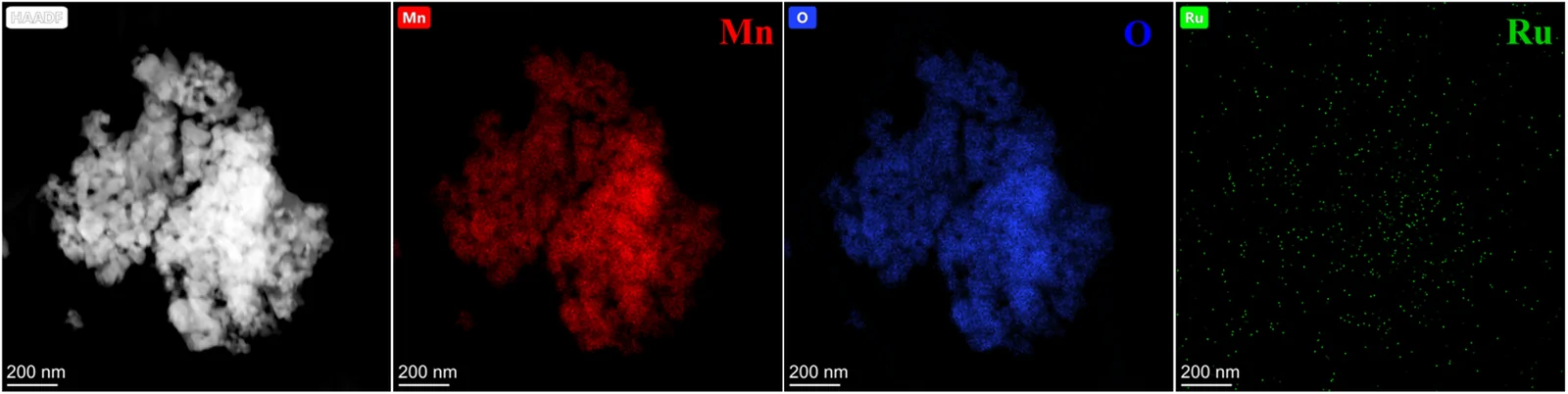
Elemental mapping and distribution of Mn, O, and Ru in Ru-Mn_2_O_3_ prepared with urea at 500 °C for 45 minutes.

TEM observations were conducted to examine the morphology and crystal structure of Ru-Mn_2_O_3_-U. As indicated in Fig. 1c, Ru-Mn_2_O_3_-U exhibits a particle morphology with a relatively uniform particle size ranging from 20 to 25 nm. In addition, an interplanar spacing of 0.38 nm is clearly visible in Fig. 1f (an enlarged version of Fig. 1e), corresponding to the (211) crystal plane of cubic Mn_2_O_3_.^40^ Additionally, Fig. 2 confirms the uniform distribution of ruthenium over Mn_2_O_3_.

**Fig. 3:**
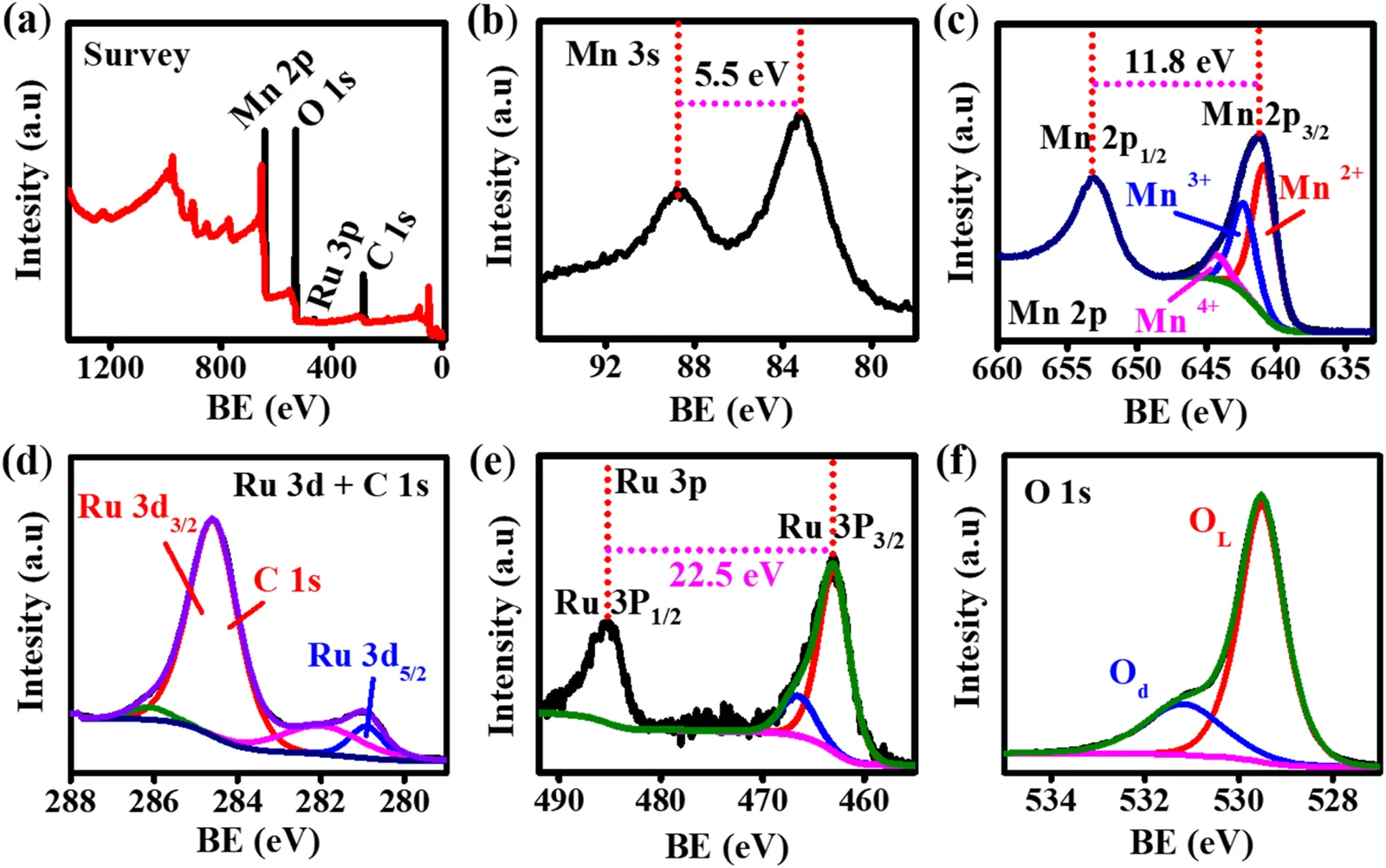
High-resolution XPS spectra of Ru-Mn_2_O_3_ prepared with urea at 500 °C for 45 minutes: (a) survey spectra, (b) Mn 3s, (c) Mn 2p, (d) Ru 3d + C 1s, (e) Ru 3p and (f) O 1s.

The surface composition of Ru-Mn_2_O_3_-U is analyzed using XPS data (Fig. 3). The oxidation number of manganese was generally determined by examining the Mn 2p and Mn 3s peaks, either individually or together. The assessment of the manganese oxidation state using the Mn 2p peak can vary significantly due to its complex structure.^41–43^ In the literature, the energy separation due to spin–orbit coupling between the Mn 2p and Mn 3s peak levels is utilized to accurately identify the oxidation state of manganese.^44^ The Mn 2p spectrum exhibits doublet peaks at 641.3 eV and 653.1 eV, which are attributed to the Mn 2p_3/2_ and Mn 2p_1/2_ states, respectively. The high-resolution XPS Mn 2p_3/2_ spectrum was deconvoluted into three distinct peaks located at 640.9 eV, 642.5 eV, and 644.2 eV, corresponding to Mn^2+^, Mn^3+^, and Mn^4+^ ions, respectively. The percentage concentrations of Mn^2+^, Mn^3+^, and Mn^4+^ are found to be 56.84%, 33.94%, and 9.23%, respectively. It is reported that the comproportionation of Mn^2+^ and Mn^4+^ produces Mn^3+^ at pH levels above 9, which is the active species for the OER.^45^ Additionally, the Mn 3s spectrum reveals peaks at 88.7 eV and 83.2 eV, with an energy separation (Δ*E*) of 5.5 eV. The average oxidation state (AOS) of Mn is calculated using the formula AOS = 8.956–1.126 × Δ*E*, resulting in a value of 2.763. It has been reported that solution combustion synthesis typically results in a large surface-to-volume ratio, which makes the formation of oxygen vacancies energetically favorable.^46^ This phenomenon stabilizes Mn^2+^ ions, leading to a decrease in the average oxidation state of manganese.

The formation of oxygen vacancies and the reduction of Mn^3+^ to Mn^2+^ can be represented as follows:
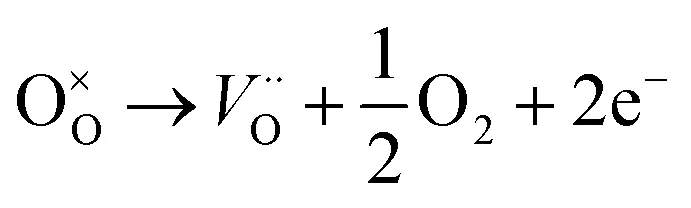
Mn^3+^ + e^−^ → Mn^2+^

As a result, the average oxidation state of manganese drops below +3.^47^ Furthermore, the presence of oxygen defects is confirmed through electron paramagnetic resonance (EPR) measurements. The EPR spectrum (Fig. S1) displays a characteristic resonance signal with a *g*-factor of 2.0398, attributed to paramagnetic centers associated with oxygen vacancies.^48^ In the O 1s spectrum, peaks at 529.5 eV and 531.1 eV are assigned to oxygen bonded to the metal (O_L_) and lattice oxygen defects (O_d_), respectively. The percentage concentrations of O_L_ and O_d_ are found to be 74.74% and 25.26%, respectively. As illustrated in Fig. 3d, the Ru 3d spectrum shows two peaks at binding energies of 284.6 eV and 280.9 eV, which correspond to Ru 3d_3/2_ and Ru 3d_5/2_ of Ru^4+^ (RuO_2_), respectively. Notably, the Ru 3d_3/2_ peak overlaps with the C 1s region at 284.6 eV, aligning with previously reported findings for RuO_2_.^49^ Moreover, the Ru 3p peaks presented in Fig. 3e further confirm the presence of RuO_2_ in the prepared sample. The Ru 3p spectrum exhibits two distinct peaks at approximately 485.6 eV and 463.1 eV, with a binding energy difference of 22.5 eV, corresponding to Ru 3p_1/2_ and Ru 3p_3/2_, respectively.^50^ Additionally, the Ru 3p_3/2_ peak can be deconvoluted into two components: the peak at 463.1 eV is attributed to Ru^4+^ oxide (RuO_2_), while the peak at 466.5 eV corresponds to RuO_2_ bound with lattice water.^51^

### 3.2 Electrochemical characterization and electrochemical activity of Ru-Mn_2_O_3_-U towards the OER in a three electrode cell

**Fig. 4:**
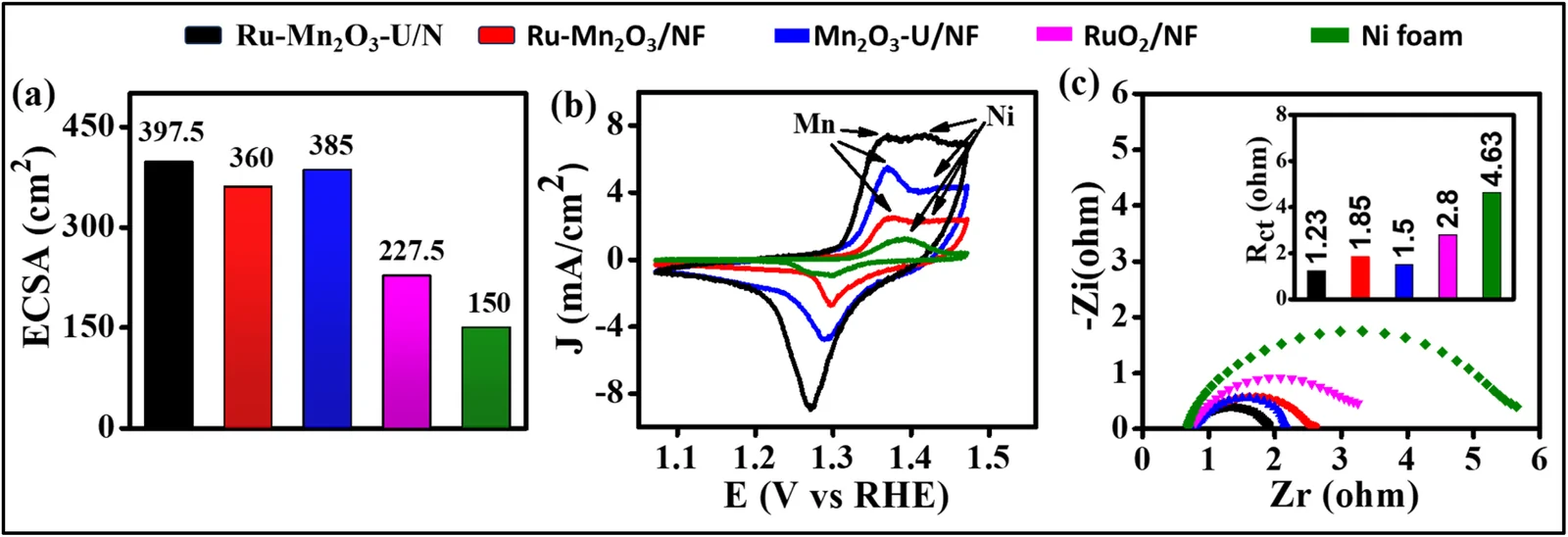
(a) ECSA, (b) CV, and (c) EIS for Ru-Mn_2_O_3_-U/NF, Mn_2_O_3_-U/NF, Ru-Mn_2_O_3_/NF, RuO_2_/NF, and Ni-foam.

As we know, the sluggish kinetics and high theoretical potential of the anodic OER significantly hinder the energy efficiency of overall water electrolysis.^52^ To enhance the kinetics and reduce the potentials in the water-splitting reaction, electrocatalysts with improved surface-active sites and low *R*_ct_ are essential. The ECSA indicates the number of active sites available for reactions, while *R*_ct_ reflects the electron transfer rate at the electrode/electrolyte interface.^53^ Fig. S2 shows the CV curves at different scan rates, along with the Δ*J versus* scan rate plots. As seen in Fig. 4a, the Ru-Mn_2_O_3_-U/NF electrode has a *C*_dl_ of 15.9 mF cm^−2^, leading to an ECSA of 397.5 cm^2^, which is higher than the 15.4 mF cm^−2^*C*_dl_ and 385 cm^2^ ECSA of the Mn_2_O_3_-U/NF electrode. Additionally, Ru-Mn_2_O_3_ was also synthesized without urea under the same experimental conditions to assess the effect of urea on *C*_dl_ and ECSA. The XRD pattern of Ru-Mn_2_O_3_ prepared without urea shows a cubic phase Mn_2_O_3_ [JCPDS 71 636] along with a tetragonal MnO_2_ phase [JCPDS 81-2261], as illustrated in Fig. S3. The Ru-Mn_2_O_3_/NF prepared without urea exhibits a *C*_dl_ and an ECSA of 14.4 mF cm^−2^ and 360 cm^2^, respectively. These results indicate that the combined effects of urea and Ru addition during synthesis enhance the ECSA of Mn_2_O_3_. The increased ECSA in the presence of urea can be attributed to the rapid exothermic decomposition of urea during synthesis, which generates large amounts of CO_2_, N_2_, and H_2_O gases. This process helps prevent particle densification, while Ru addition provides additional active sites. Moreover, the *C*_dl_/ECSA values for RuO_2_/NF and Ni-foam are found to be 9.1 mF cm^−2^/227.5 cm^2^ and 6 mF cm^−2^/150 cm^2^, respectively.

The CV technique was employed to evaluate the electrocatalytic performance of the catalyst in water electrolysis. The Ru-Mn_2_O_3_-U/NF catalyst exhibited a significantly higher redox peak current compared to the other electrodes, with an oxidation peak current of 6.9 mA cm^−2^ (Fig. 4b). This increased redox peak current suggests a more efficient generation of active species, which improves the adsorption and transfer of reaction intermediates during the OER.^54^ The charge transfer kinetics at the interface between the electrode and electrolyte for all the electrode materials were assessed using EIS. Nyquist plots for different electrodes and the Randles equivalent circuit model are shown in Fig. 4c and S4, respectively. A lower *R*_ct_ value indicates quicker electron transfer kinetics and improved electrocatalytic performance. The results presented in Fig. 4c demonstrate that Ru-Mn_2_O_3_-U/NF has a low *R*_ct_ value of 1.23 Ω. In comparison, the *R*_ct_ values for Mn_2_O_3_-U/NF, Ru-Mn_2_O_3_/NF, RuO_2_/NF, and Ni-foam are 1.5 Ω, 1.85 Ω, 2.8 Ω, and 4.63 Ω, respectively. This indicates that Ru-Mn_2_O_3_-U/NF exhibits enhanced electron transfer kinetics.

**Fig. 5:**
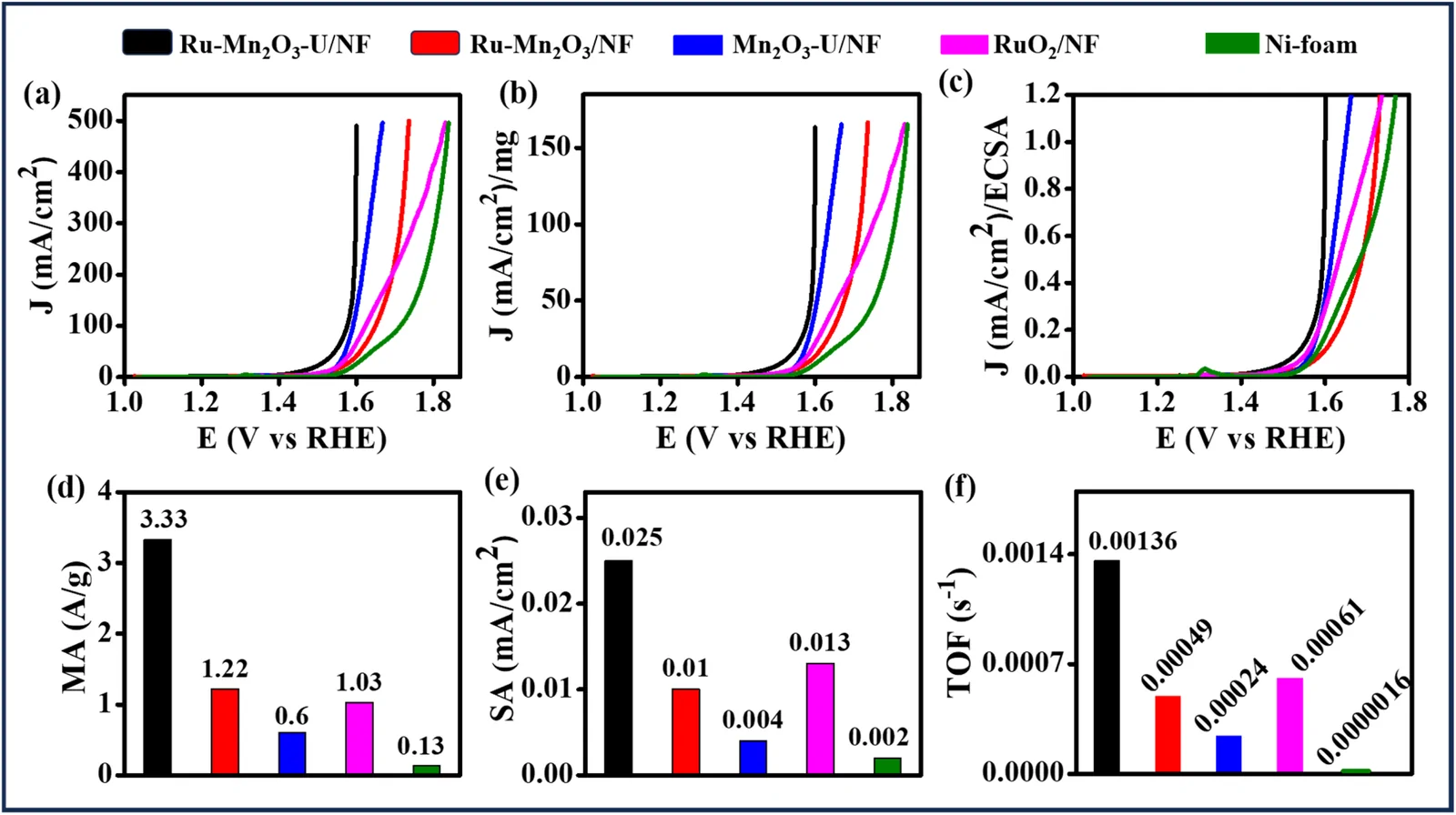
(a) LSV curves, (b) mass-normalized LSV curves, (c) ECSA-normalized LSV curves, and (d–f) the corresponding mass activity, specific activity, and turnover frequency of Ru-Mn_2_O_3_-U/NF, Mn_2_O_3_-U/NF, Ru-Mn_2_O_3_/NF, RuO_2_/NF, and Ni foam.

Fig. 5a–c illustrates the LSV curves and the corresponding mass and ECSA normalized curves for Ru-Mn_2_O_3_-U/NF, Mn_2_O_3_-U/NF, Ru-Mn_2_O_3_/NF, RuO_2_/NF, and Ni foam, all documented in 6 M potassium hydroxide electrolyte. The iR correction (85%) of the polarization curves was performed using the solution resistance (*R*_s_) obtained from electrochemical impedance spectroscopy (EIS), as detailed in Table S2. Ru-Mn_2_O_3_-U/NF requires an anodic potential of 1.44 V *vs.* RHE (210 mV) to reach 10 mA cm^−2^. In comparison, the anodic potentials required for Mn_2_O_3_-U/NF, Ru-Mn_2_O_3_/NF, RuO_2_/NF, and Ni foam are 1.52 V (290 mV), 1.52 V (290 mV), 1.51 V (280 mV), and 1.56 V (330 mV) *vs.* RHE, respectively, for the same current density.

Mn_2_O_3_ and RuO_2_ were mixed at a concentration of 0.1% to create a physically blended Ru-Mn_2_O_3_-U electrocatalyst (Ru-Mn_2_O_3_-U-PB). As shown in Fig. S5, the performance of the physically blended Ru-Mn_2_O_3_-U-PB catalyst was inferior to that of the synthesized Ru-Mn_2_O_3_-U. This suggests that the superior activity of Ru-Mn_2_O_3_-U is due to the uniform distribution and interfacial interactions established during the synthesis process, as well as an enhanced distribution of Ru species throughout the catalyst. The OER results observed for Ru-Mn_2_O_3_-U/NF demonstrate a significant improvement compared to the performance of Ru and Mn-based electrocatalysts, as highlighted in Table S4. Fig. 5d shows that Ru-Mn_2_O_3_-U/NF has the highest mass activity of 3.33 A g^−1^ at 1.44 V *versus* RHE, which is approximately 5.5 times greater than that of Mn_2_O_3_-U/NF (0.60 A g^−1^). Additionally, it surpasses Ru-Mn_2_O_3_/NF (1.226 A g^−1^) by about 2.72 times, RuO_2_/NF (1.03 A g^−1^) by 3.23 times, and Ni foam (0.13 A g^−1^) by 25.6 times. Fig. 5e shows that Ru-Mn_2_O_3_-U/NF has an OER-specific activity of 0.025 mA cm^−2^, which is 6.25 times higher than that of Mn_2_O_3_-U/NF, 2.5 times higher than that of Ru-Mn_2_O_3_/NF, 1.92 times higher than that of RuO_2_/NF, and 12.5 times higher than that of nickel foam. To further assess the intrinsic activity, the turnover frequency (TOF) of Ru-Mn_2_O_3_-U/NF was calculated at 1.44 V *vs.* RHE. Detailed calculations for the TOF are provided in the SI. As shown in Fig. 5f, Ru-Mn_2_O_3_-U/NF outperformed all other prepared samples, with a calculated TOF value of 0.00136 s^−1^. This value is 5.66 times higher than the TOF of Mn_2_O_3_-U/NF, which is 0.00024 s^−1^.

Additionally, it is approximately 2.77 times higher than the TOF of Ru-Mn_2_O_3_/NF (0.00049 s^−1^), 2.22 times higher than that of RuO_2_/NF (0.00061 s^−1^), and 850 times higher than that of nickel foam (0.0000016 s^−1^).

**Scheme 1:**
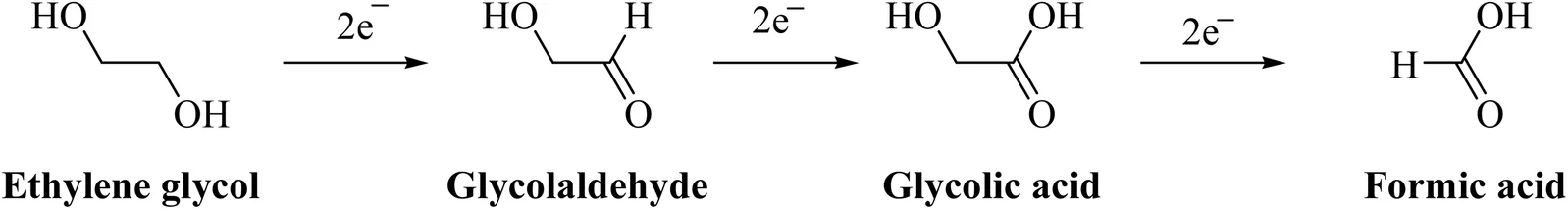
Schematic representation of the EGEOR for the formation of formic acid as a product.

In the mechanism of the oxygen evolution reaction in alkaline media, as illustrated in Scheme 1, M represents the active sites on the catalyst surface. The intermediate species that are adsorbed on these active sites are labeled as M–OH, M–O, and M–OOH. The Tafel slope values reported for the reaction steps (1), (2), and (3) are 120 mV dec^−1^, 60 mV dec^−1^, and 30 mV dec^−1^, respectively.^55,56^ Fig. S6 reveals the Tafel slope values for Ru-Mn_2_O_3_-U/NF, Mn_2_O_3_-U/NF, Ru-Mn_2_O_3_/NF, RuO_2_/NF, and Ni foam, which are 145, 150, 146, 135, and 155 mV dec^−1^, respectively. These results indicate that the initial adsorption of OH^−^, along with the first electron transfer (M + OH^−^ → M − OH + e^−^), is the rate-determining step. However, the slightly higher Tafel slope values may suggest the presence of additional kinetic limitations, such as surface heterogeneity, uncompensated resistance, or mass transport effects.^57,58^

### 3.3 Electrochemical characterization and electrochemical activity of Ru-Mn_2_O_3_-U towards the EG assisted water splitting reaction in a three electrode cell

Despite Ru-Mn_2_O_3_-U/NF showing a low overpotential for the OER, its performance remains inadequate for efficient practical hydrogen production. Moreover, the oxygen produced during anodic behavior has limited value. In contrast, the EGEOR has the capability to produce valuable industrial chemicals.^59,60^ Additionally, the EGEOR has a theoretical oxidation potential of 0.57 V *vs.* RHE and a Gibbs free energy of 5.4 kJ mol^−1^, which is anticipated to reduce energy consumption.^5,61^ To enhance hydrogen production efficiency, replacing the OER with the EGEOR can effectively lower the overpotential of the anodic reaction and improve water splitting kinetics. The feasibility of the EGEOR on Ru-Mn_2_O_3_-U/NF is examined by monitoring oxidation current, the *C*_dl_ value, and open circuit potential (OCP).

**Fig. 6:**
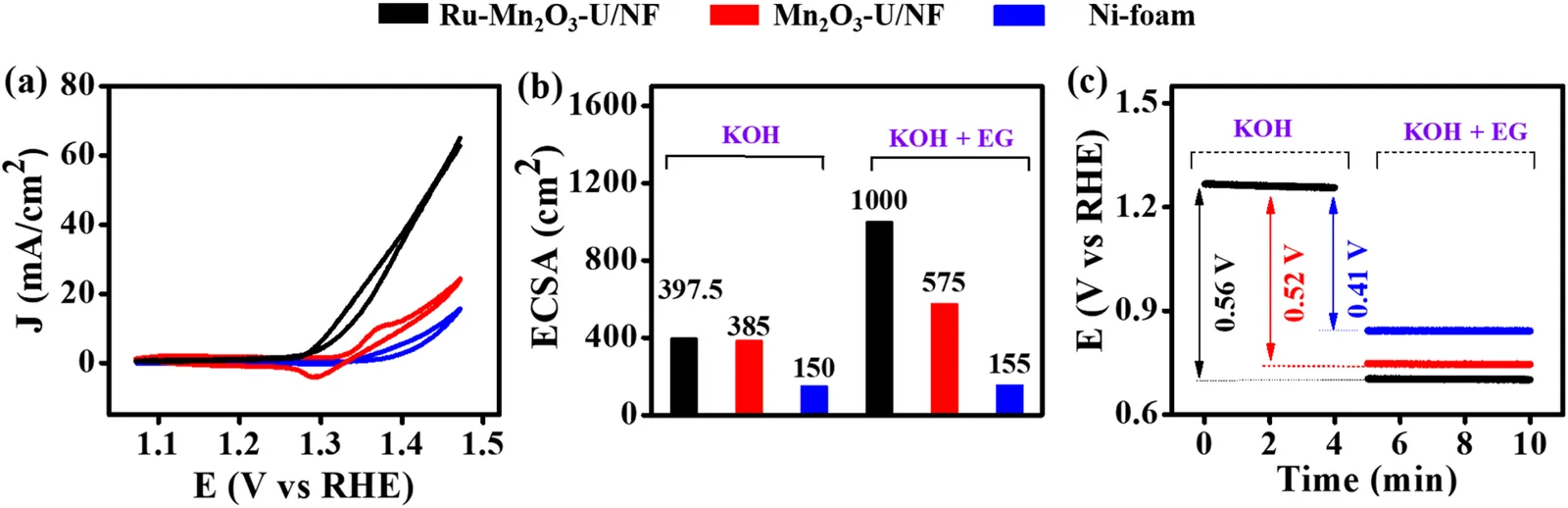
(a) CV recorded in the presence of ethylene glycol, (b) ECSA, and (c) OCP recorded with and without ethylene glycol for Ru-Mn_2_O_3_-U/NF, Mn_2_O_3_-U/NF, and Ni-foam.

The electrocatalytic activity of Ru-Mn_2_O_3_-U/NF toward the EGEOR is initially evaluated in a conventional three-electrode cell, and the results are compared with the electrochemical activity of Mn_2_O_3_-U/NF and nickel foam. As demonstrated in Fig. 6a, the Ru-Mn_2_O_3_-U/NF electrode delivers an impressive maximum current density of 65 mA cm^−2^ at 1.47 V *vs.* RHE. Remarkably, this is 2.70 times higher than the current density (24 mA cm^−2^) of Mn_2_O_3_-U/NF and 4.33 times greater than that of nickel foam, which only achieves 15 mA cm^−2^. These compelling results are further corroborated by a notable increase in the *C*_dl_ in the presence of EG, as illustrated in Fig. S7. After adding 0.4 mol L^−1^ of EG, the *C*_dl_ for Ru-Mn_2_O_3_-U/NF surges from 15.9 mF cm^−2^ to an impressive 40 mF cm^−2^. Similarly, the *C*_dl_ values for Mn_2_O_3_-U/NF and nickel foam show significant improvement, exhibiting 23 mF cm^−2^ and 7.2 mF cm^−2^, respectively. The ECSA values derived from *C*_dl_ are shown in Fig. 6b, indicating that Ru-Mn_2_O_3_-U/NF exhibits greater activity for the EGEOR.^62^

The accumulation of EG and OH^−^ is a crucial preliminary stage in the EGEOR under alkaline conditions. Therefore, higher concentrations of EG and OH^−^ near the surface of the electrocatalyst are beneficial for accelerating the EGEOR process.^63,64^ The OCP measures the adsorption of EG on the surface of electrocatalysts, providing insights into the adsorbates within the inner Helmholtz layer.^65^Fig. 6c shows a significant decrease in the OCP for the Ru-Mn_2_O_3_-U/NF electrocatalyst in the presence of EG, with a change of *Δ* = 0.56 V. In comparison, the OCP for Mn_2_O_3_-U/NF is *Δ* = 0.52 V, and for nickel foam, it is *Δ* = 0.44 V. This trend suggests that EG is favorably adsorbed onto the Ru-Mn_2_O_3_-U/NF electrocatalyst.

**Fig. 7:**
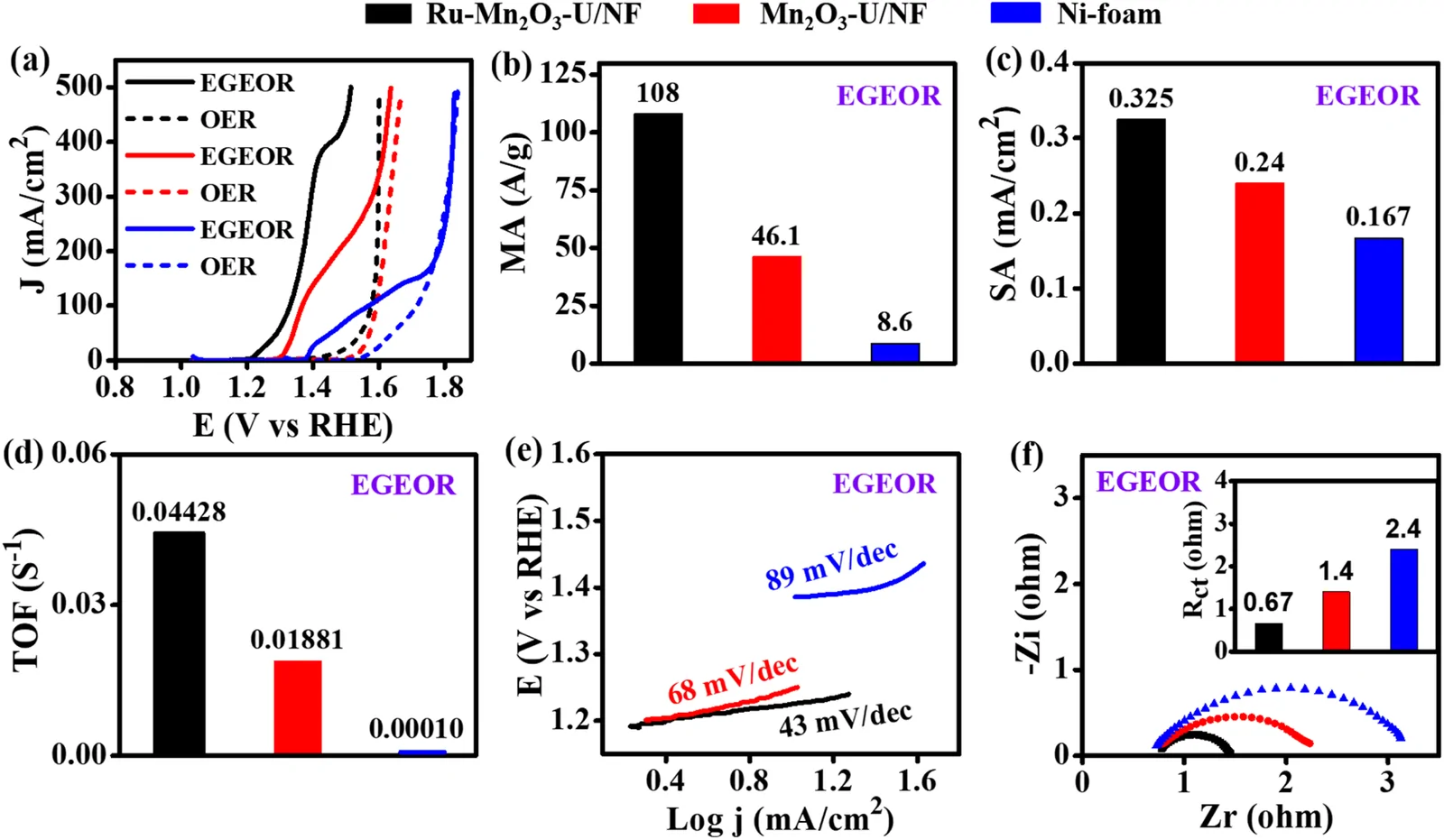
(a) LSV curves recorded in 6 M KOH-400 mmol EG, and the corresponding (b) mass activity, (c) specific activity, (d) turnover frequency, (e) Tafel plots, and (f) EIS for Ru-Mn_2_O_3_-U/NF, Mn_2_O_3_-U/NF, and Ni-foam.

The LSV curves for Ru-Mn_2_O_3_-U/NF, Mn_2_O_3_-U/NF, and Ni foam, measured in a 6 M KOH solution with ethylene glycol, are presented in Fig. 7a and S8. The Ru-Mn_2_O_3_-U/NF catalyst stands out with the lowest onset potential of 1.22 V *versus* the reversible hydrogen electrode (RHE) at an optimal EG concentration of 0.4 mol L^−1^. This value significantly surpasses the OER onset potential of 1.44 V *vs.* RHE. In comparison, the onset potentials for both Mn_2_O_3_-U/NF and nickel foam are higher, recorded at 1.30 V *vs.* RHE and 1.38 V *vs.* RHE, respectively (refer to Fig. 7a). The combination of this exceptionally low onset potential alongside a substantial increase in current density for Ru-Mn_2_O_3_-U/NF underscores the critical importance of incorporating ruthenium for enhancing the efficiency of EG oxidation. The Ru-Mn_2_O_3_-U/NF electrocatalyst requires a potential of 1.22 V *vs.* RHE at a current density of 10 mA cm^−2^, 1.32 V *vs.* RHE at 100 mA cm^−2^ and 1.45 V at 400 mA cm^−2^ in a 6 M KOH solution with 400 mmol of EG. In contrast, the Mn_2_O_3_-U/NF and Ni-foam substrates demonstrate significantly higher potentials at equivalent current densities (Fig. 7a). As shown in Fig. 7b, Ru-Mn_2_O_3_-U/NF demonstrates impressive electrocatalytic activity for the electrochemical ethylene glycol oxidation (EGEOR), achieving a mass activity of 108.47 A g^−1^ at 1.40 V *versus* RHE. This performance is approximately 2.35 times greater than that of Mn_2_O_3_-U/NF, which achieves a mass activity of 46.1 A g^−1^, and is 12.6 times higher than nickel foam's 8.6 A g^−1^ at the same potential. In Fig. 7c, the specific activities for the EGEOR at a potential of 1.40 V *vs.* RHE illustrate a clear advantage: Ru-Mn_2_O_3_-U/NF delivers 0.325 mA cm^−2^, overshadowing Mn_2_O_3_-U/NF at 0.240 mA cm^−2^ and nickel foam at 0.167 mA cm^−2^. Additionally, Fig. 7d illustrates the turnover frequency at 1.40 V *versus* RHE, highlighting the effectiveness of Ru-Mn_2_O_3_-U/NF with a turnover rate of 0.04427 s^−1^. In comparison, Mn_2_O_3_-U/NF shows a lower rate of 0.01881 s^−1^, while nickel foam has an even smaller rate of just 0.00010 s^−1^. These compelling data demonstrate the superior potential of Ru-Mn_2_O_3_-U/NF in advancing electrocatalytic applications.

Ru-Mn_2_O_3_-U/NF exhibits a Tafel slope value of 43 mV dec^−1^ (Fig. 7e), which is significantly lower than its OER Tafel slope of 145 mV dec^−1^. Additionally, the Tafel slope values for Mn_2_O_3_-U/NF and nickel foam are measured at 68 mV dec^−1^ and 89 mV dec^−1^, respectively. In Fig. 7f, it is observed that the Ru-Mn_2_O_3_-U/NF electrode has the lowest *R*_ct_ value of 0.67 Ω. In comparison, the *R*_ct_ values for the Mn_2_O_3_-U/NF and Ni-foam electrodes are 1.4 Ω and 2.4 Ω, respectively. To further emphasize the role of urea in the synthesis process, we evaluated the electrochemical performance of a urea-free control sample (Ru-Mn_2_O_3_/NF) as seen in Fig. S9. This sample required a potential of 1.59 V *versus* RHE at 100 mA cm^−2^, exhibiting a Tafel slope of 73 mV dec^−1^ and a charge transfer resistance (*R*_ct_) value of 1.6 Ω. These findings indicate that Ru-Mn_2_O_3_/NF displays inferior electrochemical activity toward the EGEOR compared to the Ru-Mn_2_O_3_-U/NF sample synthesized with urea. In contrast, the Ru-Mn_2_O_3_-U/NF electrode demonstrates more favorable and rapid reaction kinetics for the EGEOR.

The oxidation products of EG are analyzed using ^1^H NMR spectral data. The data presented in Fig. S10 confirm that glycolate is the major product, while formate is the minor product. A schematic representation of the EGEOR is shown in Scheme 1. In summary, EG is first oxidized to glycolaldehyde, which is subsequently converted to glycolic acid and formate.^66,67^

The faradaic efficiency (FE) of glycolate and formate was evaluated based on the ^1^H NMR data, following established methods in the literature.^68^ The calculation details are provided in the supporting information. The highest FE observed was 91.8% for glycolate and 1.85% for formate, achieved at a potential of 1.32 V *vs.* RHE, highlighting the effectiveness of this approach.

### 3.4 Electrocatalytic activity of Ru-Mn_2_O_3_-U towards the EG assisted water electrolysis reaction in a two electrode cell

The enhanced performance of Ru-Mn_2_O_3_-U in the EGEOR has resulted in its application as a bifunctional electrode material in the construction of an EG-based conventional membrane-less electrolyzer, Ru-Mn_2_O_3_-U/NF‖Ru-Mn_2_O_3_-U/NF (Fig. S11). In an EG-6 M KOH electrolyte, a membrane-less conventional electrolyzer requires the following potentials: 1.52 V, 1.63 V, and 1.69 V at 298 K to achieve currents of 10 mA cm^−2^, 50 mA cm^−2^, and 100 mA cm^−2^, respectively.

Additionally, when tested at an industrial water electrolysis temperature of 348 K,^4^ the membrane-less electrolyzer demonstrates the following potentials: 1.44 V, 1.58 V, and 1.62 V at 10 mA cm^−2^, 50 mA cm^−2^, and 100 mA cm^−2^, respectively. In contrast, significantly higher cell potentials are observed at these current densities when using a 6 M KOH electrolyte.

Additionally, a single-stack cell has been fabricated to demonstrate its potential for practical applications using an anion exchange membrane (Fig. S12). The stack cell shows significantly improved performance in water-splitting compared to the traditional membrane-less cell. This stack cell requires specific cell potentials to achieve various currents at different temperatures. At 298 K, the required potentials are 1.51 V for 10 mA cm^−2^, 1.59 V for 50 mA cm^−2^, and 1.59 V for 100 mA cm^−2^. In contrast, at 348 K, the minimum potentials needed are lower: 1.38 V for 10 mA cm^−2^, 1.52 V for 50 mA cm^−2^, and 1.54 V for 100 mA cm^−2^. Table 1 summarizes the results of the membrane-less conventional and single stack electrolyzers operated at 298 K and 348 K.

**Table 1:** Comprehensive evaluation of the water splitting activity of membrane-less conventional electrolyzers and stacked electrolyzers

EG-assisted water electrolyzer	E @ 10 mA cm^−2^		E @ 50 mA cm^−2^		E @ 100 mA cm^−2^	
	298 K	348 K	298 K	348 K	298 K	348 K
Conventional membrane less cell (1 cm × 1 cm)	1.52	1.44	1.63	1.58	1.69	1.62
Stack cell (2.2 cm × 2.2 cm)	1.51	1.38	1.59	1.52	1.59	1.54

The results observed for the Ru-Mn_2_O_3_-U/NF electrocatalyst demonstrate a significant performance improvement compared to both precious and non-precious metal-based electrocatalysts, as highlighted in Table S5. This suggests strong potential for enhancing efficiency in electrochemical applications. The liberated hydrogen gas is collected using the water drainage method for a stack cell that operates at room temperature with a current of 20.8 mA cm^−2^, allowing us to calculate the faradaic efficiency (FE). The Ru-Mn_2_O_3_-U/NF‖Ru-Mn_2_O_3_-U/NF stack cell exhibits a FE of approximately 93%.

**Fig. 8:**
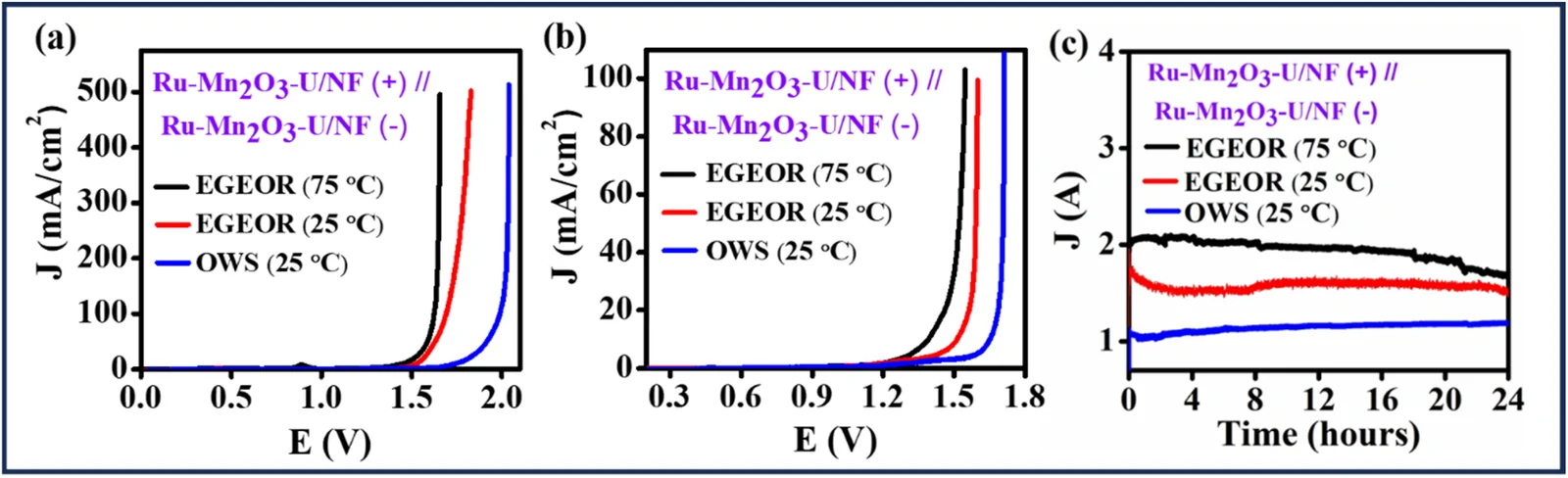
LSV curves of (a) the membrane-less conventional single cell, (b) the single stack cell with an anion membrane, and (c) stability curves recorded in the stack cell.

Furthermore, the Ru-Mn_2_O_3_-U/NF‖Ru-Mn_2_O_3_-U/NF stack cell, depicted in Fig. 8c, exhibits operational stability over 24 hours, maintaining a constant current without any noticeable decay. Post-stability XPS characterization confirms that the presence of Mn and Ru, along with their characteristic binding energies, remains nearly unchanged compared to those of the fresh catalyst (refer to Fig. S13). This indicates that the surface chemical composition of the catalyst is well-preserved.

The electrical energy consumption for hydrogen production *via* OWS and EG-WSR has been calculated using an empirical formula:
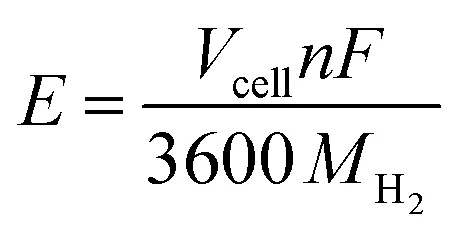
where *V*_cell_ = operating cell voltage, *n* = 2 electrons per mole of H_2_, *F* = 96 485 C mol^−1^, and *M*_H_2__ = 0.002 kg mol^−1^. By substituting the relevant values into this equation, it simplifies to*E*_kWh kg^−1^H_2__ = 26.8 × *V*_cell_

The energy consumption for OWS and EG-WSR is 45.2 kWh kg^−1^ H_2_ and 42.6 kWh kg^−1^ H_2_, respectively, indicating that Ru-Mn_2_O_3_-U/NF could serve as an effective bifunctional electrocatalyst.

## 4 Conclusion

We achieved a uniform distribution of ruthenium within Mn_2_O_3_ using a simple one-pot urea-assisted redox reaction. This method improves the electrochemical surface area, charge-transfer kinetics, and catalytic activity by evenly distributing ruthenium and preventing particle densification. The ruthenium-loaded Mn_2_O_3_ nanoparticles demonstrate excellent performance in the EGEOR, achieving mass and specific activities of 108.4 A g^−1^ and 0.325 mA cm^−2^, respectively. Notably, the combination of the EGEOR and water splitting requires a minimum cell potential of 1.38 V at 10 mA cm^−2^, making hydrogen production cost-effective. These results highlight Ru-Mn_2_O_3_-U as a promising, energy-efficient electrocatalyst for sustainable hydrogen production and chemical synthesis.

## Conflicts of interest

The authors have declared no conflicts of interest.

## Supplementary Material

NA-OLF-D6NA00300A-s001

## Data Availability

Data are available from authors upon reasonable request. Supplementary information (SI): comprehensive details on the structural characterization of the electrocatalyst, its performance, and product analysis. See DOI: https://doi.org/10.1039/d6na00300a.
