# Supplementary material for: Organic substrate oxidation-driven water electrolysis enabled by Ru-loaded Mn2O3 nanoparticles for energy-efficient hydrogen generation
Source: Nanoscale Adv. 2026 Sep 14. Online ahead of print. doi: 10.1039/d6na00300a (PMC13573632; doi:10.1039/d6na00300a)
Supplement: Supplementary file 1 [file NA-OLF-D6NA00300A-s001.pdf]

Supplementary Information (SI) for Nanoscale Advances.
This journal is © The Royal Society of Chemistry 2026

Supporting Information

Organic Substrate Oxidation-Driven Water Electrolysis Enabled by Ru-Loaded Mn_2O_3 Nanoparticles for Energy-Efficient Hydrogen Generation

P.D. Sachin[1,2], Ashoka S[1,2]*, M. Madesh Kumar[3], K. Yogesh[4]

[1]*Department of Chemistry, School of Applied Sciences, REVA University, Bengaluru-560064, India*

[2]*Center for Nano & Functional Materials, REVA Research Center, REVA University, Bengaluru-560064, India*

[3]*Department of Physics, School of Applied Sciences, REVA University, Bengaluru-560064, India*

[4]*Department of Physics, School of Engineering, Dayananda Sagar University, Bengaluru-562112, India*

Synthesis of Ru-Mn_2O_3-U-PB

Manganese nitrate (oxidizer) and urea (fuel) are reacted in a 1:1 ratio. Subsequently, thermal treatment is performed at 500 °C for 45 minutes, leading to the formation of Mn_2O_3 and physical mixing of Ru with 0.1% ruthenium oxide (RuO_2) to form PB-Ru- Mn_2O_3-U

Characterization

X-ray diffraction (XRD) patterns were determined using an X-ray diffractometer with Cu Kα radiation (Rigaku Ultima IV X-ray diffractometer). PerkinElmer Fourier transform infrared spectrophotometer was employed to examine the samples within the range of 4000-400 cm^{-1}. The morphologies and elemental composition were observed by transmission electron microscopy (TEM) using Talos 200FG2 transmission electron microscope (TEM). X-ray photoelectron spectroscopy (XPS) was recorded using the Thermo Scientific X-ray photoelectron spectroscopy with an Al Kα X-ray source. And, NMR spectroscopy was done by using a Bruker AMX 400 (100 MHz) NMR spectrometer to analyze the formaldehyde oxidation product. The electrochemical workstation used for conducting water electrolysis reactions is manufactured by Origalys France.

Calculation of ECSA by double-layer capacitance (C_{dl})

The ECSA can be calculated from the C_{dl} as follows:

$$\text{ECSA} = \frac{C_{\text{dl}}}{C_{\text{s}}}$$

where C_s is the specific capacitance of a flat standard electrode with 1 cm^2 of real surface area as 0.04 mF/cm^2.

Specific Activity (SA)

The Specific Activity (SA) was calculated by using following equation.

$$SA = \frac{j_{geo}}{ECSA}$$

j_{geo} is the current density per geometric area.

Mass Activity (MA)

The specific Mass Activity (MA) was calculated by using following equation.

$$MA = \frac{j_{geo}}{catlyst\ mass\ loading}$$

j_{geo} is the current density per geometric area.

Turn Over frequency (TOF)

The TOF was calculated by using following equation.

$$TOF = \frac{J * A}{n * F * M}$$

J is the current density at a given overpotential (A/cm^2)

A is the surface area of electrode

n is the number of electron transfer at given at given overpotential

F is the faradays constant

M is the number of moles of active material on the electrode

$$M\ \ (number\ of\ moles\ of\ active\ material) = \frac{catalyst\ mass\ loading}{molecular\ weight\ of\ the\ active\ catlyst}$$

Catalyst mass loading used for all catalyst 3 mg/cm^2, geometric surface area of electrode 1 cm^2, faradays constant 96485 C mol^{-1}, the number of electron transfer at given at given overpotential for OER is 4 and for EGEOR is 4.

Calculation For

Ru-Mn_2O_3 - U

number of moles of active material (M) = $\frac{0.003\ g}{157.74\ g}$

= 0.0000190186 mol/cm^2

The total number of moles of active martial (Ru-Mn_2O_3 – U) on the electrode calculated to be 0.0000190186 mol/cm^2. This value was used for TOF calculation assuming that all the loaded catalyst is electrochemically active.

For Ru-Mn_2O_3

number of moles of active material (M) = $\frac{0.003\ g}{157.74\ g}$

= 0.0000190186 mol/cm^2

The total number of moles of active martial (Ru-Mn_2O_3) on the electrode calculated to be 0.0000190186 mol/cm^2.

Mn_2O_3-U

number of moles of active material (M) = $\frac{0.003\ g}{157.8\ g}$

Mn_2O_3-U = 0.0000190114 mol/cm^2

The total number of moles of active martial (Mn_2O_3 – U) on the electrode calculated to be 0.0000190114 mol/cm^2.

Energy efficiency

We calculated the electrical energy consumption for hydrogen production. The formula for electrical energy consumption is given by:

$$W = V_{\text{cell}} \times I \times t$$

The normalized to hydrogen production is given by,

$$E = \frac{V_{\text{cell}} nF}{3600 M_{H_2}}$$

where, V_{cell}= operating cell voltage, $n = 2$ electrons per mole of H_2, $F = 96485$ C mol^{-1}, and $M_{H_2} = 0.002$ kg mol^{-1}. By substituting the relevant values into this equation, it simplifies to:

$$E_{\text{kWh/kg } H_2} = 26.8 \times V_{\text{cell}}$$

Table S1. Comparison of the energy consumption for overall water splitting and ethylene glycol-assisted water splitting

**Parameter**	**Overall water splitting**	**EG-assisted water splitting**
Cell voltage	1.69 V at 100 mA/cm^2	1.59 V at 100 mA/cm^2
Energy consumption	45.2 kWh kg^{-1} H_2	42.6 kWh kg^{-1} H_2

The overall water splitting requires 45.2 kWh kg^{-1} H_2 and ethylene glycol-assisted water splitting requires 42.6 kWh kg^{-1} H_2.

Faradic efficiency

A known amount of an internal standard (DMSO) was added to each electrolyte sample prior to analysis. The concentrations of glycolate and formate were determined by comparing the integrated peak areas of their characteristic proton signals with that of the internal standard, according to the following equation:

$$C_{product} = \frac{A_{product}/n_{product}}{A_{IS}/n_{IS}} * C_{IS}$$

where $A_{product}$ and A_{IS} are the integrated peak areas of the product and the internal standard, respectively; $n_{product}$ and n_{IS} represent the number of equivalent protons contributing to the selected resonances; and C_{IS} is the known concentration of the DMSO internal standard.

C_{IS} concentration of internal standard = 0.0017 moles

A_{IS} integrated peak area of glycolate is 3

A_{IS} integrated peak area for formate is 0.02

n_{IS} is the integrated peak area of internal standard 4.5

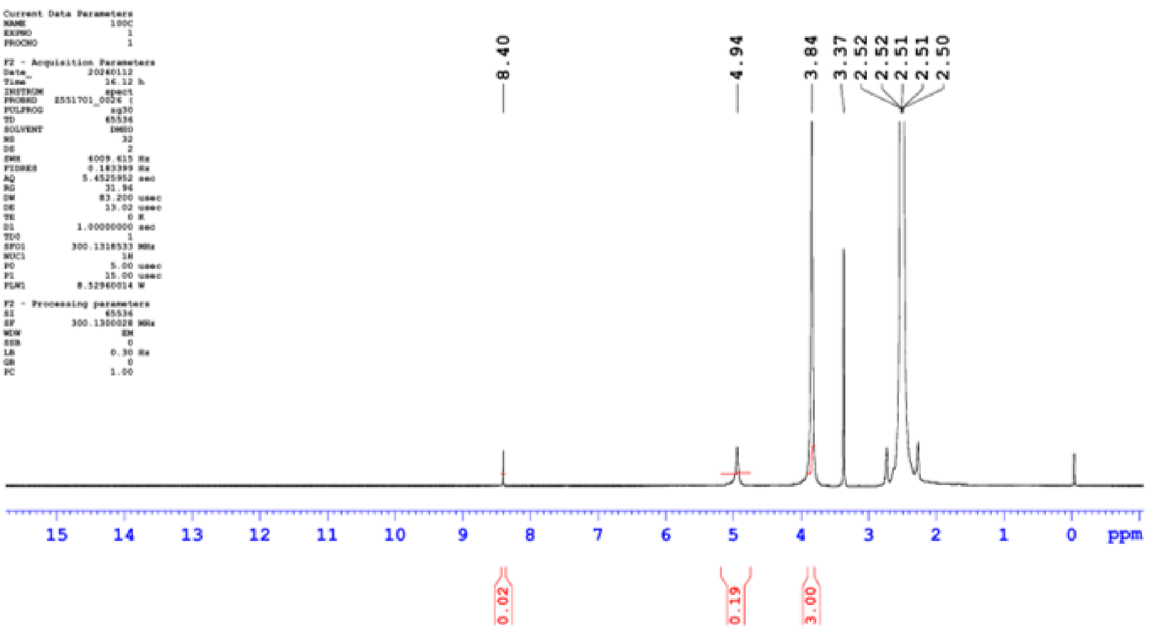

^{1}H NMR spectrum of ethylene glycol oxidized product

Faradic efficiency of glycolate

$$C_{product} = \frac{3}{4.5} * \frac{6}{2} * 0.0017$$

$$C_{product} = 0.0034254 \text{ moles}$$

Concentration of glycolate formed = 0.0034254 moles

Faradic efficiency is calculated by using equation

$$\text{Faradic efficiency} = \frac{N.F.n}{Q} * 100$$

N is the number of electrons required for electrocatalytic oxidation of EG to glycolate (4e), and where n refers to moles of product formed.

Faradic efficiency = $$\frac{4 * 96485 * 0.0034254}{1440} * 100$$

Faradic efficiency of glycolate = 91.8 %

Faradic efficiency of formate

$$C_{product} = \frac{0.02}{4.5} * \frac{6}{1} * 0.0017$$

$C_{product}$ = 0.00004613 moles

Concentration of formate formed = 0.00004613 moles

Faradic efficiency is calculated by using equation

Faradic efficiency = $$\frac{n.F.N}{Q} * 100$$

N is the number of electrons required for electrocatalytic oxidation of EG to formate (6e), and where n refers to moles of product formed.

Faradic efficiency = $$\frac{6 * 96485 * 0.00004613}{1440} * 100$$

Faradic efficiency of Formate formed = 1.85 %

Experimental details of FE for H_2 by two electrode measurements

We integrated two electrode electrolyzers to determine FE for H_2 evolution by separating an ion exchange membrane. 100 mL of KOH + 0.4 mol/L ethylene glycol electrolyte solution was taken in the anode compartment and then the cell was sealed without a leakage. The electrolyte solution in the working compartment was constantly stirred (200 rpm) during the electrolysis. A constant current is applied (20.8 mA/cm^2) and the liberated hydrogen gas is collected using the water drainage method And the Faradic efficiency of H_2 was calculated using the below formulae.

Faradic efficiency (FE) $$= \left(\frac{n * F * N_{product}}{Q_{total}}\right) \times 100\%$$

Where, n = number of electrons involved in the reaction,

F= Faraday constant

$N_{product}$ = moles of product formed

Q_{total} = total charge passed (coulomb)

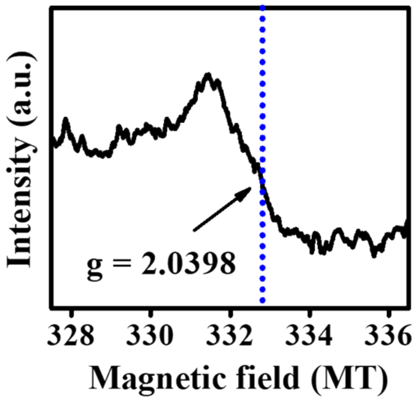

Figure S1. EPR spectrum of Ru-Mn_2O_3-U

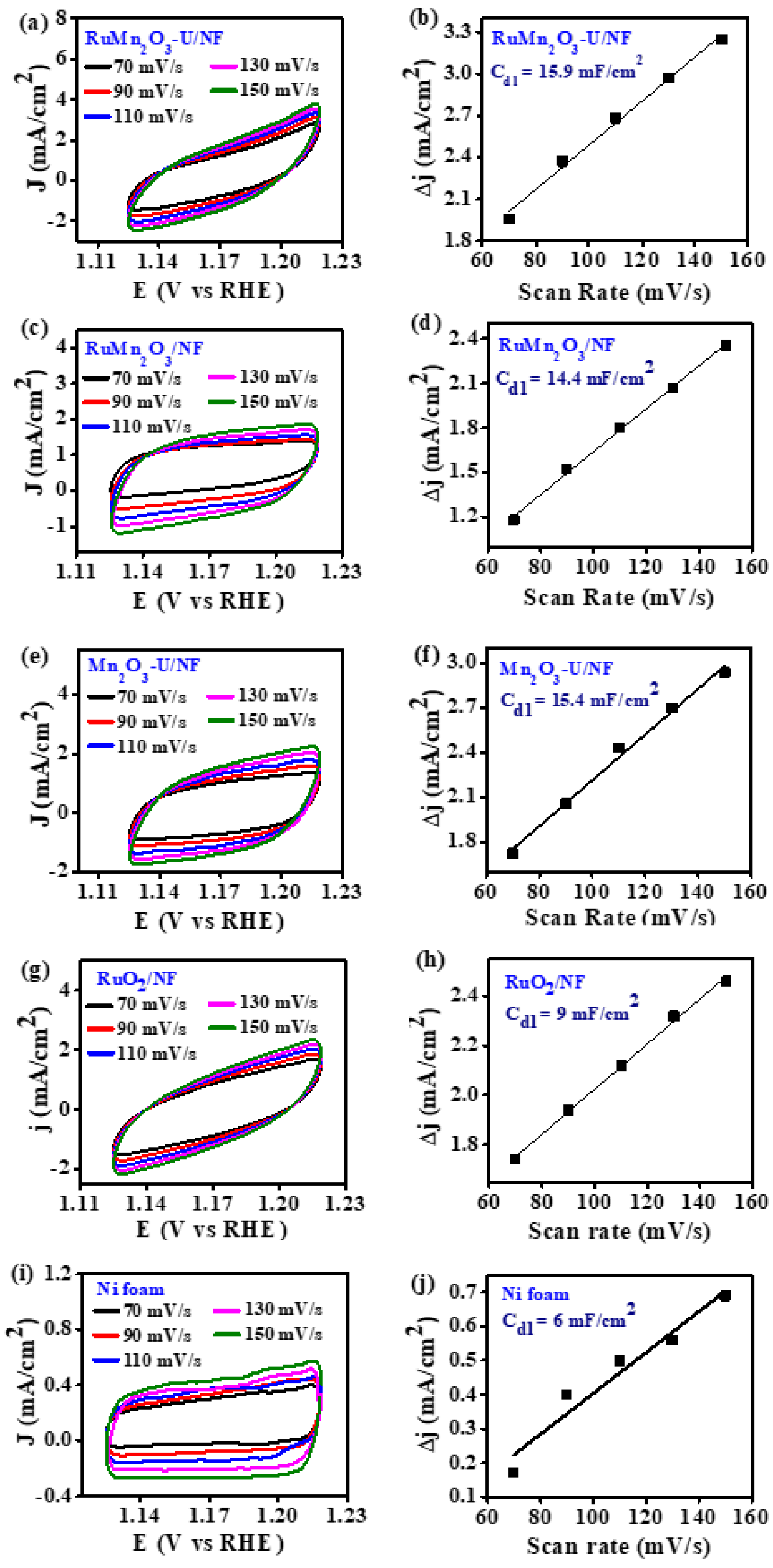

Figure S2. Cyclic voltammograms recorded in 6 M KOH at various scan rates, along with the electrochemical double layer capacitance measured for Ru-Mn_2O_3-U/NF, Mn_2O_3-U/NF, Ru-Mn_2O_3/NF, RuO_2/NF, and nickel foam.

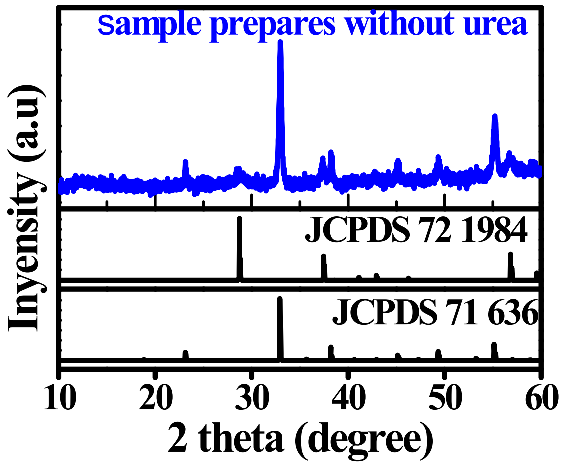

Figure S3. The powder XRD pattern of Ru-Mn_2O_3 prepared at 500 °C for 45 minutes without urea.

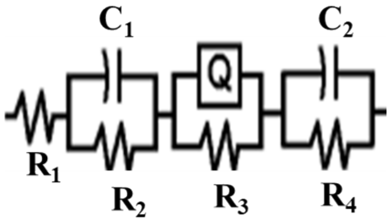

Figure S4. Equivalent electrical circuit used to fit EIS data

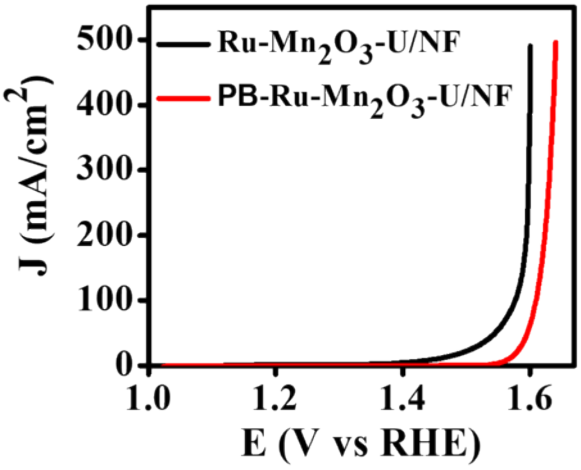

Figure S5. The Linear sweep voltammetry curves of Physical blended (PB) Ru-Mn_2O_3-U and as synthesized Ru-Mn_2O_3-U

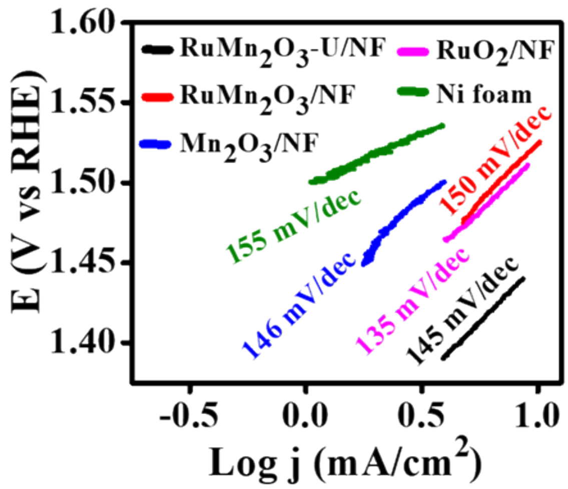

Figure S6. Tafel slope values observed in 6 M KOH for Ru-Mn_2O_3-U/NF, Mn_2O_3-U/NF, Ru-Mn_2O_3/NF, RuO_2/NF, and Ni foam

Scheme S1: - OER mechanism in alkaline electrolyte

M represents the active sites on the catalyst surface.

Step 1: OH^- adsorption

$OH^- + M \rightarrow M - OH + e^-$ Step 2: Formation of oxo species

$M - OH + OH^- \rightarrow M - O + H_2O + e^-$ Step 3: O–O bond formation (rate-determining in many cases)

$M - O + OH^- \rightarrow M - OOH + e^-$ Step 4: Oxygen release

$M - OOH + OH^- \rightarrow O_2 + H_2O + M + e^-$

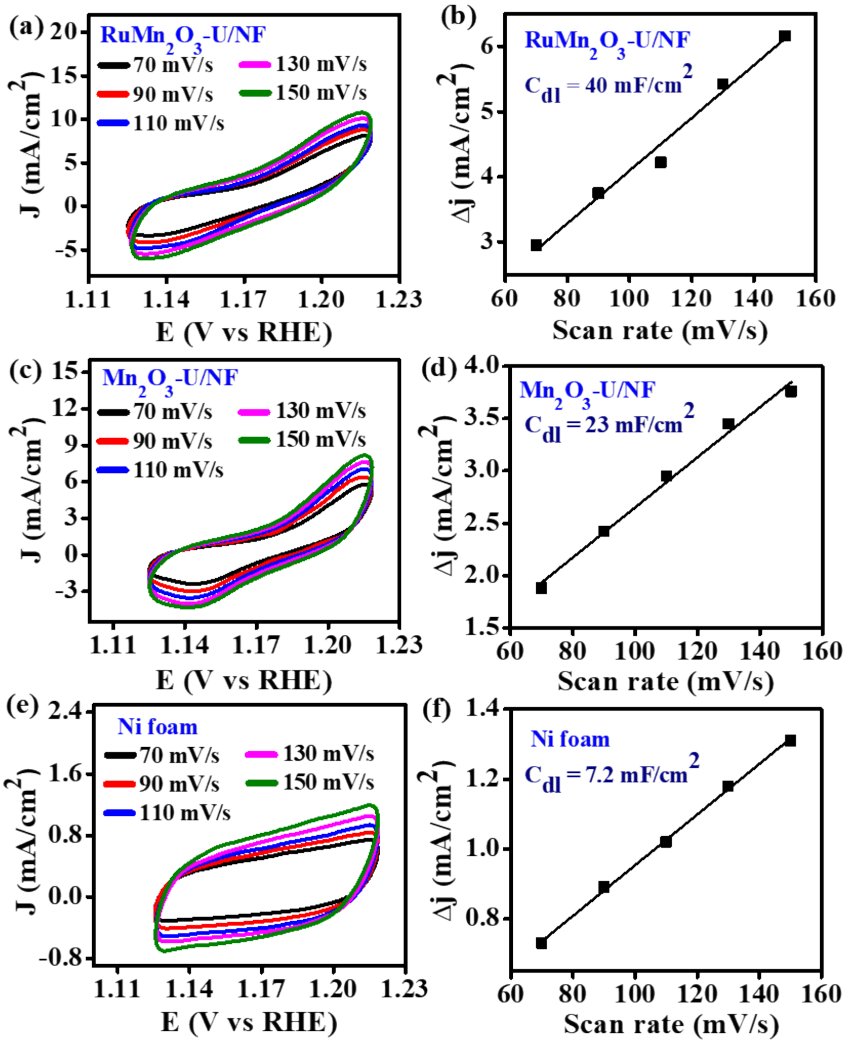

Figure S7. Cyclic voltammograms recorded in 6 M KOH- ethylene glycol at various scan rates, along with the electrochemical double layer capacitance measured for Ru-Mn_2O_3-U/NF, Mn_2O_3-U/NF and Ni foam

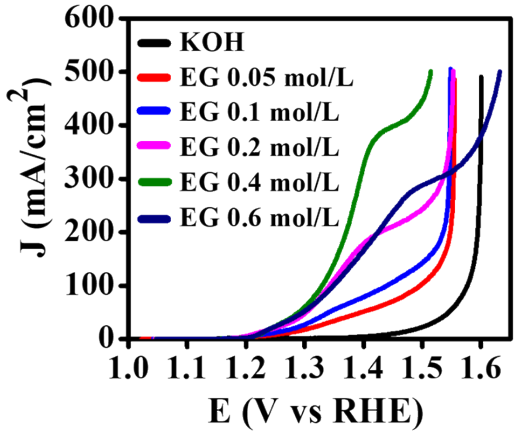

Figure S8 – LSV profiles of Ru-Mn_2O_3-U/NF, Mn_2O_3, and Ni-Foam were recorded in 6 M KOH with varying concentrations of ethylene glycol.

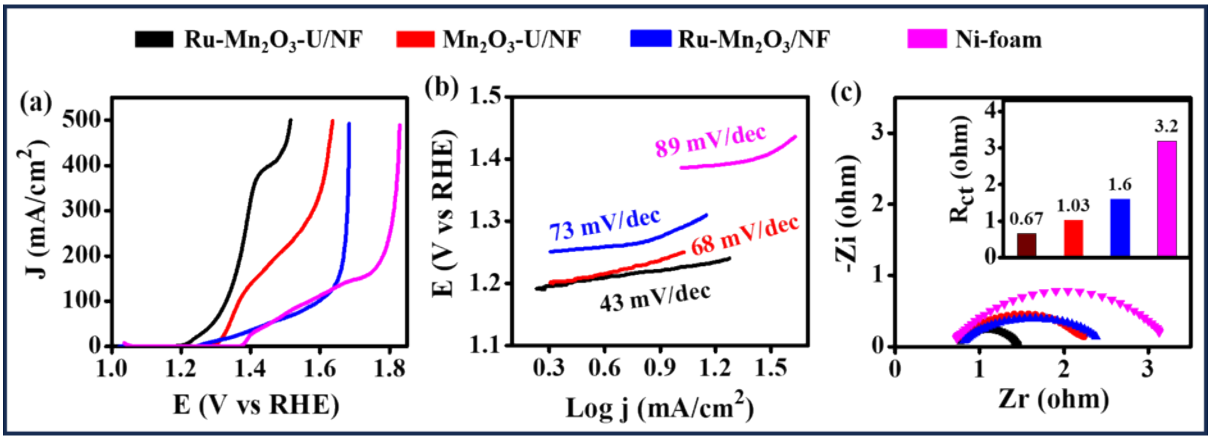

Figure S9. (a) LSV curves recorded in 6M KOH-400 mmol EG, and the corresponding, (b) Tafel plots, and (c) EIS for Ru-Mn_2O_3-U/NF, Mn_2O_3-U/NF, Ru-Mn_2O_3/NF and Ni-foam

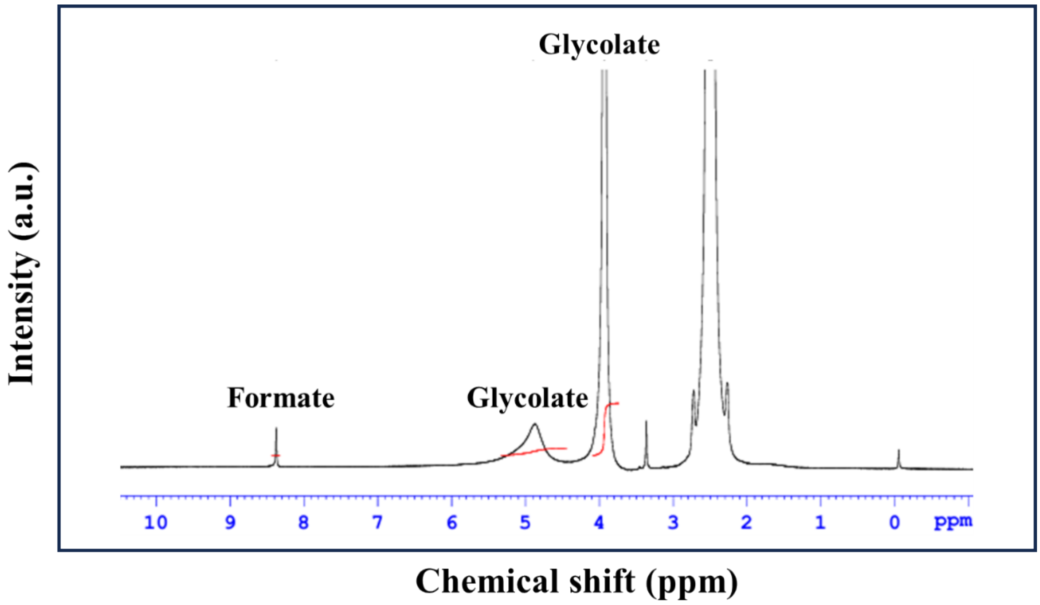

Figure S10. ^1H NMR spectrum of ethylene glycol oxidized product

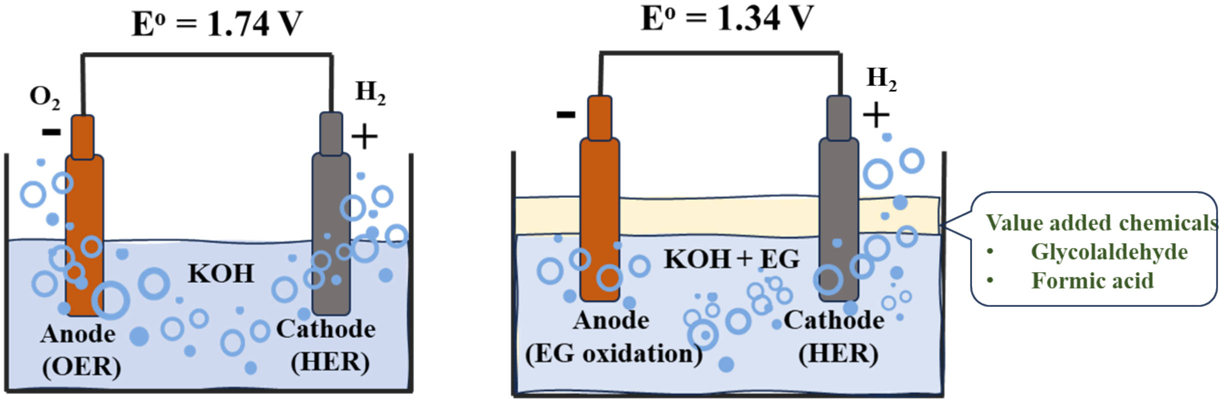

Figure S11. Schematic representation of a membrane-less conventional electrolyzer designed for a 6 M KOH solution and a 6 M KOH-Ethylene glycol mixture.

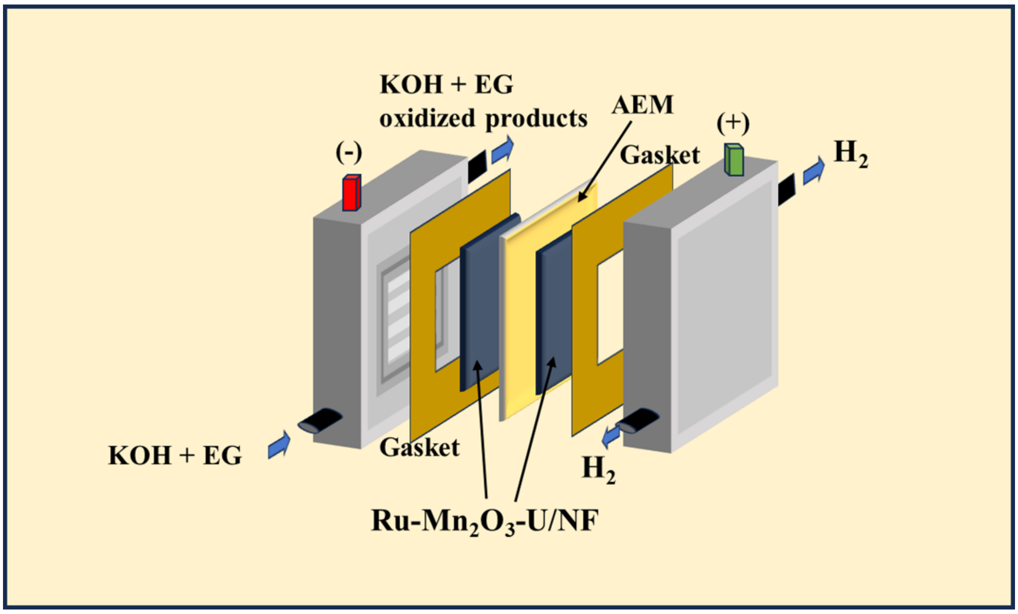

Figure S12. Schematic representation of the AEM electrolyzer designed for a 6 M KOH solution and a 6 M KOH-ethylene glycol mixture.

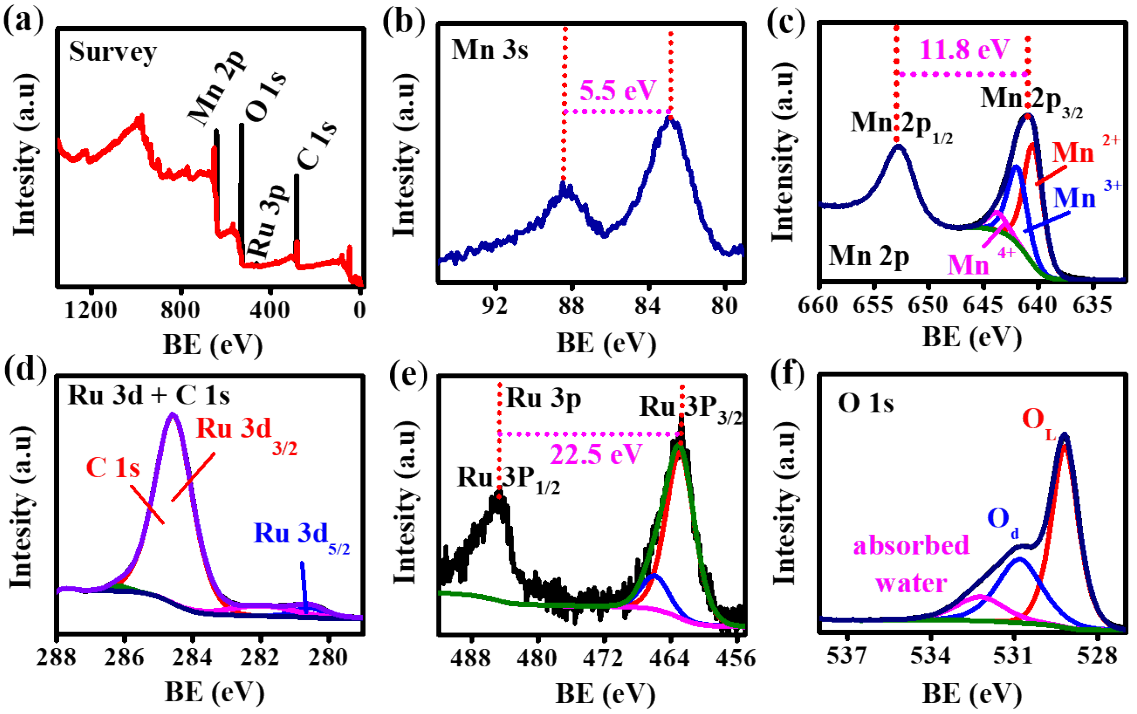

Figure S13. Post stability High-resolution XPS spectra of Ru-Mn_2O_3-U (a) Survey spectra (b) Mn 3s, (c) Mn 2p, (d) Ru 3d + C1s (e) Ru 3p and (f) O 1s.

Table S2. EIS-derived solution resistance values (R_s) for Ru–Mn_2O_3-U/NF, Ru-Mn_2O_3/NF, Mn_2O_3-U/NF, RuO_2/NF and Ni foam during OER and EGEOR.

Electrocatalysts	OER: R_s (Ω)	EGEOR: R_s (Ω)
**Ru-Mn_2O_3-U/NF**	0.76	0.77
**Mn_2O_3-U/NF**	0.76	0.79
**Ni-foam**	0.68	0.72

Tabel S3. Double-layer capacitance (C_{dl}) and electrochemically active surface area (ECSA) derived from cyclic voltammetry measurements.

Electrocatalysts	OER		EGEOR	
	C_{dl} (mF/cm^2)	ECSA (cm^2)	C_{dl} (mF/cm^2)	ECSA (cm^2)
**Ru-Mn_2O_3-U/NF**	15.9	397.5	40	1000
**Mn_2O_3-U/NF**	15.4	385	23	575
**Ni-foam**	6	150	6.2	155

Tabel S4. Comparison of the performance of Ru-Mn_2O_3-U in the oxygen evolution reaction with findings from the literature.

Electrocatalyst	Preparation methods and conditions	Overpotential	Substrate and electrolyte	Ref
Present work (Ru-Mn_2O_3-U/NF)	Solution combustion method	210 mV @ 10 mA/cm^{-2}	Ni-foam, 6 M KOH	
Present work Physical blended (Ru-Mn_2O_3-U/NF)	Solution combustion method fallowed by physical blending	330 mV @ 10 mA/cm^{-2}	Ni-foam, 6 M KOH	
Ru-RuO_2 /C-X.	Magnetic induction heating method.	240 mV @ 10 mA/cm^{-2}	Glassy carbon, 1 M KOH	[3]
RuO_2/Ru-CNFs-350	Facile electrospinning technique	203 mV @ 10 mA/cm^{-2}	RDE Glassy carbon, 1 M KOH	[4]
Ru–CoO_x	Via facile ion exchange in aqueous Ru^{3+} solution, and transformation to a unique hollow (Ru-Co)O_x nanosheet via annealing at 300 °C in air,	171.2 mV @ 10 mA/cm^{-2}	Carbon cloth, 1 M KOH	[5]

Pd−Ru	Facile synthesis by treating processors at 135 °C for 20 h under 2.5 bar H_2	225 mV @ 10 mA/cm^{-2}	1 M KOH	6
Ru_2Ni_2 SNs/C	Ultrasonicated for around 30 min, and heated at 170 °C for 5 h in an oil bath	357 mV @ 10 mA/cm^{-2}	RDE Glassy carbon, 1 M KOH	7
Ru@FeP_4/Fe_2PO_5	Heat treatment coupling electrodeposition strategy	249 mV @ 100 mA/cm^{-2}	RDE Glassy carbon, 1 M KOH	8
MnFeRu-90	Hydrothermal synthesis at 150 °C for 10 h, then calcined at 600 °C for 6 h	180 mV @ 10 mA/cm^{-2}	Ti plates	9
Ru–MnFeP/NF	Hydrothermal synthesis then phosphatized by $NaH_2PO_2 \cdot H_2O$	191 mV @ 20 mA/cm^{-2}	Ni-foam, 1 M KOH	10
NS-MnO_2	facile one-step hydrothermal treatment	320 mV at 10 mA cm^{-2}	Ni-foam	11
Fe-MnO_2/NF	facile one-step hydrothermal treatment	330 mV at 20 mA cm^{-2}	Ni-foam, 1 M KOH	12

Tabel S5. Comparison of the performance of Ru-Mn_2O_3 towards ethylene glycol assisted water splitting reaction with findings from the literature.

**Electrocatalyst**	**Preparation methods and conditions**	**Overpotential**	**Substrate and electrolyte**	**Ref**
Present work (Ru-Mn_2O_3-U/NF)	Solution combustion method	1.59 V @ 100 mA/cm^{-2}	6.0 M KOH + 0.4 M EG, Ni-foam	
h-PtAg-NW	In situ dynamic evolution method	0.63 V @ 100 mA/cm^{-2}	1.0 M KOH + 1.5 M EG, carbon fibre	13
$NiSe_2$	Preparation of Ni–Se precursor then thermally decomposed in the presence of a mixture of solvents/surfactants	1.395 V @ 50 mA/cm^{-2}	1.0 M KOH + 1 M EG, glassy carbon	14
M-PtBiMo IMSs	One-pot wet chemical method	0.60 V @ 100 mA/cm^{-2}	1.0 M KOH + 1 M EG	15
Pd DNT	Electrochemical deposition method	1.1 V @ 4 mA/cm^{-2}	0.1 M $NaClO_4$ + EG, glassy carbon	16

$Ru/Ni_2P/NF$	Cyanogel-$NaBH_4$ method and succedent phosphating process.	1.37 V @ 100 mA/cm^{-2}	1.0 M KOH + 0.5 M EG, Ni-foam	[17]
AuPd@Pd	One-step wet-chemical co-reduction method using allantoin as a structure-directing agent	0.53 V @ 10 mA/cm^{-2}	0.5 M KOH + 0.5 M EG	[18]
($Co(OH)_2$-Cl/NF)	Pre catalyst, Cl^- doped $Co(OH)_2$ on Ni foam	1.37 V @ 400 mA/cm^{-2}	1.0 M KOH + EG, Ni-foam	[19]
Fe(0.3 wt%)-CoPO	One-pot hydrothermal synthesis	1.426 V @100 mA/cm^{-2}	1 M KOH + 1 M EG, Ni-foam	[20]
$Ni(OH)_2/NF$	In-situ self-growth on the surface of Ni foam by facile acid etching method	1.60 V @ 500 mA/cm^{-2}	1 M KOH + 1 M EG, Ni-foam	[21]
Au-Pd/C	Precipitation method and dried under vacuum for 24 h	0.9 V @ 400 mA/cm^{-2}	2 M KOH + 2 M EG, Corbon cloth	[22]

References

1 Z. Li, Y. Yan, S.-M. Xu, H. Zhou, M. Xu, L. Ma, M. Shao, X. Kong, B. Wang, L. Zheng and H. Duan, *Nat. Commun.*, 2022, **13**, 147.

2 G. F. Pauli, B. U. Jaki and D. C. Lankin, *J. Nat. Prod.*, 2005, **68**, 133–149.

3 D. Pan, B. Yu, J. Tressel, S. Yu, P. Saravanan, N. Sangoram, A. Ornelas-Perez, F. Bridges and S. Chen, *Advanced Science*, DOI:10.1002/advs.202414534.

4 M. Zhong, S. Yan, J. Xu, C. Wang and X. Lu, *Inorg. Chem. Front.*, 2022, **9**, 4881–4891.

5 C. Wang and L. Qi, *Angew. Chem. Int. Ed.*, 2020, **59**, 17219–17224.

6 L. Gloag, T. M. Benedetti, S. Cheong, C. E. Marjo, J. J. Gooding and R. D. Tilley, *J. Am. Chem. Soc.*, 2018, **140**, 12760–12764.

7 J. Ding, Q. Shao, Y. Feng and X. Huang, *Nano Energy*, 2018, **47**, 1–7.

8 W. Xia, M. Ma, X. Guo, D. Cheng, D. Wu and D. Cao, *ACS Appl. Mater. Interfaces*, 2023, **15**, 44827–44838.

9 Y. Wu, M. Tariq, W. Q. Zaman, W. Sun, Z. Zhou and J. Yang, *ACS Omega*, 2020, **5**, 7342–7347.

10 D. Chen, Z. Pu, R. Lu, P. Ji, P. Wang, J. Zhu, C. Lin, H. Li, X. Zhou, Z. Hu, F. Xia, J. Wu and S. Mu, *Adv. Energy Mater.*, DOI:10.1002/aenm.202000814.

11 Y. Zhao, C. Chang, F. Teng, Y. Zhao, G. Chen, R. Shi, G. I. N. Waterhouse, W. Huang and T. Zhang, *Adv. Energy Mater.*, DOI:10.1002/aenm.201700005.

12 J. Lu, H. Wang, Y. Sun, X. Wang, X. Song and R. Wang, *Chemical Engineering Journal*, 2021, **417**, 127894.

13 Y. Li, Q. Liao, P. Ji, S. Jie, C. Wu, K. Tong, M. Zhu, C. Zhang and H. Li, *JACS Au*, 2025, **5**, 714–726.

14 J. Li, L. Li, X. Ma, X. Han, C. Xing, X. Qi, R. He, J. Arbiol, H. Pan, J. Zhao, J. Deng, Y. Zhang, Y. Yang and A. Cabot, *Advanced Science*, DOI:10.1002/advs.202300841.

15 X. Yang, Q. Yuan, T. Sheng and X. Wang, *Chem. Sci.*, 2024, **15**, 4349–4357.

16 Y. Lum, J. E. Huang, Z. Wang, M. Luo, D.-H. Nam, W. R. Leow, B. Chen, J. Wicks, Y. C. Li, Y. Wang, C.-T. Dinh, J. Li, T.-T. Zhuang, F. Li, T.-K. Sham, D. Sinton and E. H. Sargent, *Nat. Catal.*, 2020, **3**, 14–22.

17 G. Ma, N. Yang, Y. Xue, G. Zhou and X. Wang, *ACS Appl. Mater. Interfaces*, 2021, **13**, 42763–42772.

18 Q. Liu, Y.-R. Xu, A.-J. Wang and J.-J. Feng, *Int. J. Hydrogen Energy*, 2016, **41**, 2547–2553.

19 D. X. Yang, J. J. He, H. G. Xu, J. K. Liu, Q. Niu, H. G. Yang and P. F. Liu, *Small*, DOI:10.1002/smll.202512718.

20 X. Bai, J. Lin, B. Cheng, C. Yang, X. Wang, Y. Li, T. Ishida, T. Murayama, G. Xiu and M. Lin, *J. Catal.*, 2026, **455**, 116708.

21 K. Liu, Y. Wang, F. Liu, C. Liu, R. Shi and Y. Chen, *Chemical Engineering Journal*, 2023, **473**, 145292.

22 H. A. Miller, M. Bellini, F. Vizza, C. Hasenöhrl and R. D. Tilley, *Catal. Sci. Technol.*, 2016, **6**, 6870–6878.
